# Single Cell Multi‐Omics Revealing the Important Role of MR1 Mediated MAIT Cells in Maintaining Rejection for Liver Transplantation

**DOI:** 10.1111/cpr.70194

**Published:** 2026-03-10

**Authors:** Hailun Cai, Xinqiang Li, Xin Zhou, Xueteng Wang, Zhuoyu Jia, Ruidong Ding, Yurong Luo, Ye Wang, Shipeng Li, Wenxing Sun, Dongxing Wu, Dahong Teng, Kai Zhao, Guanghui Pei, Jinzhen Cai, Bin Wu

**Affiliations:** ^1^ Organ Transplant Center Fujian Medical University Union Hospital Fuzhou China; ^2^ Organ Transplantation Center Affiliated Hospital of Qingdao University Qingdao China; ^3^ Institute of Organ Donation and Transplantation Medical College of Qingdao University Qingdao China; ^4^ Pathology Department The Affiliated Hospital of Qingdao University Qingdao China; ^5^ Department of Hepatopancreaticobiliary Surgery, Henan Provincial People's Hospital Zhengzhou University Zhengzhou China

**Keywords:** graft rejection, liver transplantation, major histocompatibility complex class I related protein‐1, mucosal‐associated invariant T cells

## Abstract

Mucosal‐associated invariant T (MAIT) cells, representing one of the most abundant subsets of unconventional T cells, have been shown to play a significant role in regulating immune responses. However, their immunoregulatory roles in the context of liver transplantation (LT) immunity remain largely undefined. To address this, we conducted single‐cell RNA/TCR sequencing, flow cytometry, and multiplex immunohistochemical (mIHC) assays to identify the proportion and characteristics of CD8+ MAIT cells in humans and mice following liver transplantation. We found that CD8+ MAIT cells were prominently represented in the single‐cell CD8 profiles of human transplanted livers, demonstrating strong signalling associations with macrophages, whilst the fractional populations of MAIT1 and MAIT17 were distinctly clustered. In parallel, the proportion of CD8+ MAIT cells was significantly elevated in mouse LT models, revealing a dynamic trend where percentages increased at 1 and 2 weeks post‐transplant, peaking at 3 weeks. Furthermore, using established MR1 knockout (MR1KO) LT mice, we observed that mice lacking MAIT cells exhibited milder rejection responses, indicating that MR1 mediates rejection by influencing the remodelling of the TCR repertoire after transplantation. Collectively, our study reveals that MAIT cells play a critical role in LT rejection, as MR1KO alleviated inflammatory responses and mitigated rejection via TCR repertoire remodelling. By mapping the dynamic changes of MAIT cells throughout the rejection process, these findings lay the groundwork for further investigations into the role of these cells in transplant immunity.

## Graphical abstract

This multi‐omics study reveals the significant role of MAIT cells in liver transplant rejection. MAIT cells drive rejection in liver transplantation via the MR1 axis. Their absence reshapes T cell responses and clonal expansion, attenuating allograft injury.

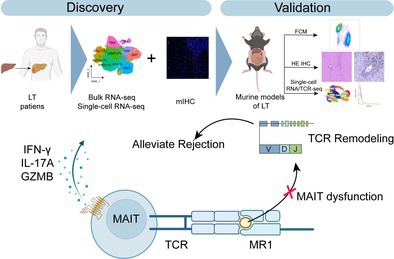

## 1 Introduction

Liver transplantation (LT) is the most effective treatment for end‐stage liver disease, and postoperative immune homeostasis greatly affects the quality of patient survival [1, 2]. After LT, the interaction and coordination between donor‐derived cells and the recipient's immune system are essential for graft acceptance [3, 4]. The transplantation immune remodelling process is complex and includes a variety of immune cells such as T cells, macrophages, dendritic cells, etc. Different immune cells are activated during the interaction, and receptor‐activated T cells recognise donor alloantigens, triggering an adaptive inflammatory immune response that leads to allograft rejection [5]. T cells and their subpopulations such as CD4 + regulatory T cells (Treg) related to immune tolerance and cytotoxic CD8 + T cells associated with transplant rejection have an important role in overall graft rejection [6]. An in‐depth understanding of the localisation and role of T cells in transplantation immunology is essential. Therapeutic strategies targeting these cells and their subpopulations, the search for cell markers and cell subpopulations is critical to the design of targeted rejection monitoring delivery strategies and therapeutic interventions, marking a major leap forward in the field of transplantation immunology [7, 8].

Mucosal‐associated invariant T (MAIT) cells are a unique subset of innate‐like T cells restricted by major histocompatibility complex class I‐related protein 1 (MR1) [9]. Unlike conventional T cells, MAIT cells express a semi‐invariant T cell receptor (TCR) α‐chain (Vα7.2 in humans) and recognise vitamin B metabolite‐based antigens [10]. Crucially, MAIT cells are preferentially enriched in the human liver, constituting up to 50% of the hepatic T cell pool, and are strategically positioned around bile ducts and portal tracts to act as a first line of defence [11, 12, 13].

Recent studies have highlighted the pivotal role of MAIT cells in various liver pathologies. Beyond their antimicrobial functions, MAIT cells can be activated via TCR‐independent pathways to secrete high levels of proinflammatory cytokines such as IL‐17A, IFN‐γ, and TNF‐α, as well as cytotoxic molecules like Granzyme B [14, 15]. Evidence suggests that activated MAIT cells contribute to tissue inflammation and fibrosis in non‐alcoholic fatty liver disease, alcoholic liver disease, and viral hepatitis [16, 17, 18, 19, 20]. Given that the majority of human MAIT cells exhibit a CD8 + CD161hi phenotype [21], aligning with the CD8+ T cell predominance observed in cellular rejection, it is plausible that these liver resident cells play a significant, yet underappreciated, role in allograft rejection.

Despite their abundance in the hepatic microenvironment and proven involvement in chronic liver diseases, the specific immunomodulatory mechanisms of MAIT cells during the acute and chronic phases of LT rejection remain largely unexplored. Our previous single‐cell atlas of human LT identified MAIT cells as a distinct subset within the changing immune landscape [22, 23]; however, their functional contribution requires further validation. In this study, we integrated single‐cell RNA sequencing (scRNA‐seq), TCR tracking, and bulk RNA sequencing (bulk RNA‐seq) with validation in an MR1‐deficient mouse model to comprehensively characterise the role of MAIT cells. We aim to fill the knowledge gap regarding MAIT cells in transplantation immunology and uncover their potential as therapeutic targets for regulating liver allograft rejection.

## 2 Results

### 2.1 Single‐Cell Transcriptomics Defines CD8 + MAIT in Human Liver Transplantation

**FIGURE 1:**
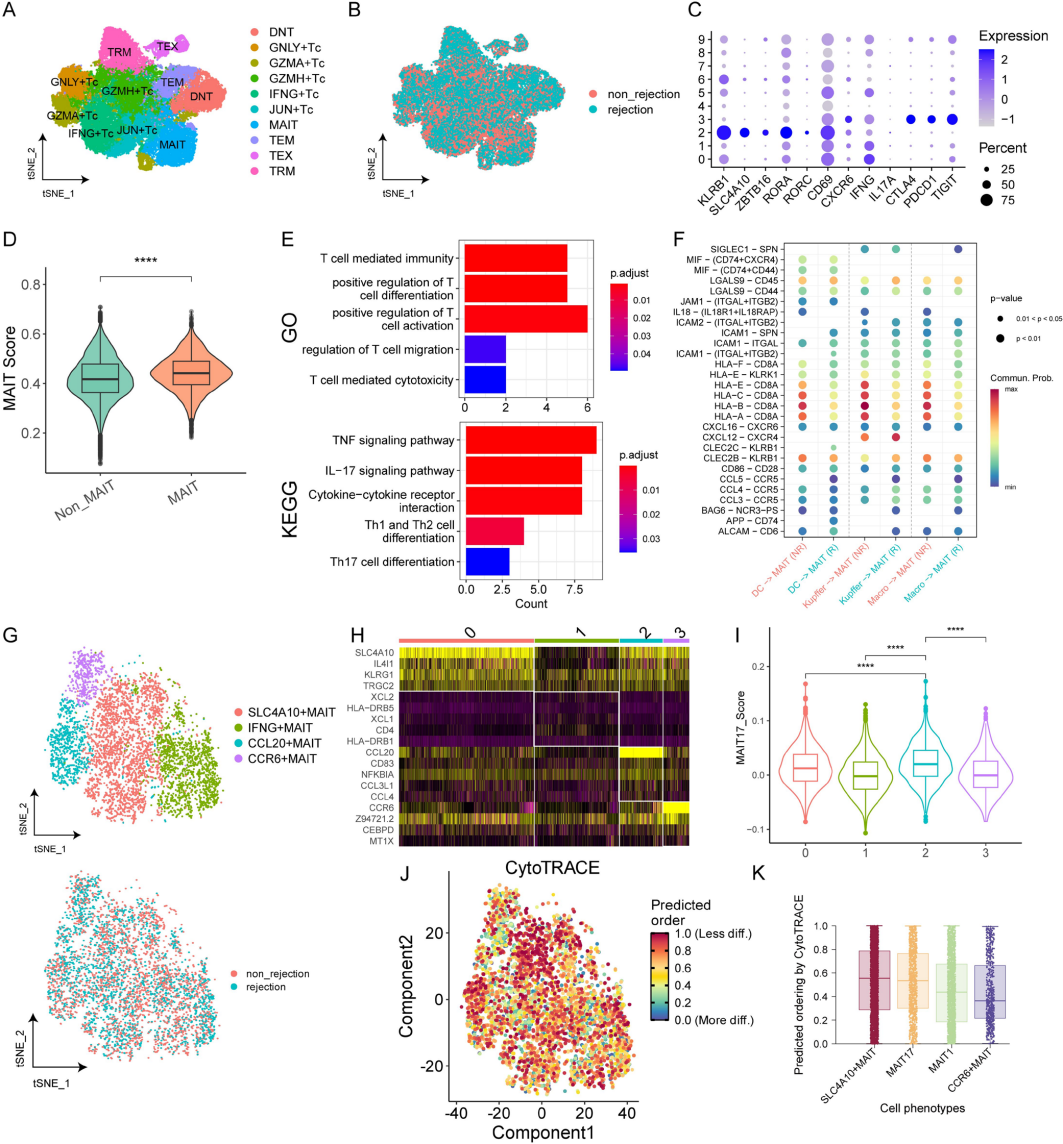
Single‐cell transcriptomic landscape of CD8+ T cells in human liver allografts. (A, B) t‐SNE visualisation of CD8+ T cell clusters (A) and their distribution in non‐rejection (NR) versus rejection (R) samples (B, C) Expression of canonical MAIT cell markers (KLRB1, SLC4A10, ZBTB16, RORA, RORC) across identified clusters. (D) Violin plot comparing MAIT signature scores between the MAIT cluster and other CD8+ T cell subsets. (E) Gene Ontology (GO) and KEGG pathway enrichment analyses of differentially expressed genes (DEGs) in the MAIT cluster. (F) Ligand‐receptor interactions between CD8+ MAIT cells and myeloid subsets in NR versus R groups. (G) Re‐clustering of the MAIT population across MAIT annotation (top) and diagnosis (down). (H) Heatmap of top DEGs across sub‐clusters. (I) Violin plot of MAIT17 signature scores. (J) TSNE shows MAIT cells were predicted with differentiation states using CytoTRACE. (K) Box plot shows the differentiation of four phenotypes.

To characterise the MAIT cells in LT human, we applied single‐cell dataset from 17 samples [24] and subset 46,117 CD8 + cells from the atlas, delineating into 10 clusters (Figure 1A). According to their functional contribution, we annotated cytotoxic T cells (Tc), mucosal‐associated invariant T cells (MAIT), tissue‐resident memory T cells (TRM), effector memory T cells (TEM), exhausted T cells (TEX), and double negative T cells (DNT). We identified five clusters of Tc, which are GLNY+T cells, GZMA+T cells, GZMH+T cells, IFNG+T cells, and JUN + T cells. The distribution analysis of CD8 + T cells indicated rejection dominance (Figure 1B). Then we annotated cluster 2 into MAIT according to the high expression of KLRB1, SLC4A10, ZBTB16, RORA, and RORC (Figure 1C). To confirm the MAIT designation, we adopted MAIT scoring [25], MAIT exhibited higher ratings proving its distinctiveness compared to other clusters (Figure 1D).

Using Gene Ontology (GO) terms and Kyoto Encyclopaedia of Genes and Genomes (KEGG) functional pathway analysis showed that differentially expressed genes (DEGs) within CD8 + MAIT comprised mainly T cell activation, differentiation, migration and immunity, cytotoxicity mediated. KEGG analysis revealed the capabilities of CD8 + MAIT were significantly involved in TNF, IL‐17 pathway, cytokine receptor interaction and Th1, Th2, Th17 differentiation (Figure 1E, Table S1). Interrogation of cellular communication networks demonstrated that MAIT participated in comprehensive discussions with myeloid cells, such as dendritic cells, Kupffer cells, and macrophages (Figures 1F, S1, Table S2). In the case of rejection, MAIT cells demonstrated incremental prevalence of signals in CXCR4, CXCR6 and HLA whilst augmented reception of TNF, IFNG and CCL5.

To study MAIT in greater depth, we identified 5615 MAIT cells (2702 for rejection and 2913 for non‐rejection) from the CD8 + T cell population, grouped them into four distinct clusters and the distribution of MAIT cells in rejection and non‐rejection samples (Figure 1G). Each cluster exhibited elevated expression of genes such as SLC4A10, IFNG, CCL20, and CCR6 (Figure 1H, Table S3). Based on the MAIT1 and MAIT17 gene sets [24], we scored the 4 groups of MAIT cells and found that the C2_CCL20+ MAIT cluster was more aligned with the MAIT17 profile (Figure 1I). Amongst MAIT1‐related genes, we observed differences between clusters C1 and C3 (Figure S1). Notably, the C2_MAIT cluster expressed high levels of IFNG, favouring a MAIT1‐like profile. Whilst earlier studies suggested an absence of distinct MAIT1 and MAIT17 clustering in humans, our analysis of liver transplant samples revealed a more pronounced trend towards these subsets.

To further investigate the developmental dynamics and lineage relationships amongst these MAIT subsets, we performed trajectory inference analyses. Using CytoTRACE to predict the differentiation potential (Figure 1J), we observed that the SLC4A10 + MAIT cluster exhibited the highest predicted ordering score compared to other subsets (Figure 1K). This suggests that SLC4A10 + MAIT cells likely represent a less differentiated, resting precursor pool. In contrast, the MAIT1 (IFNG+) and MAIT17 (CCL20+) clusters displayed lower CytoTRACE scores, indicating they are more terminally differentiated effector states. Collectively, these results suggest that SLC4A10 + MAIT cells differentiate into specialised functional MAIT1 and MAIT17 subsets within the liver transplant microenvironment.

### 2.2 Identify CD8 + MAIT Marker Expression in Different Rejection States of Human LT Livers

**FIGURE 2:**
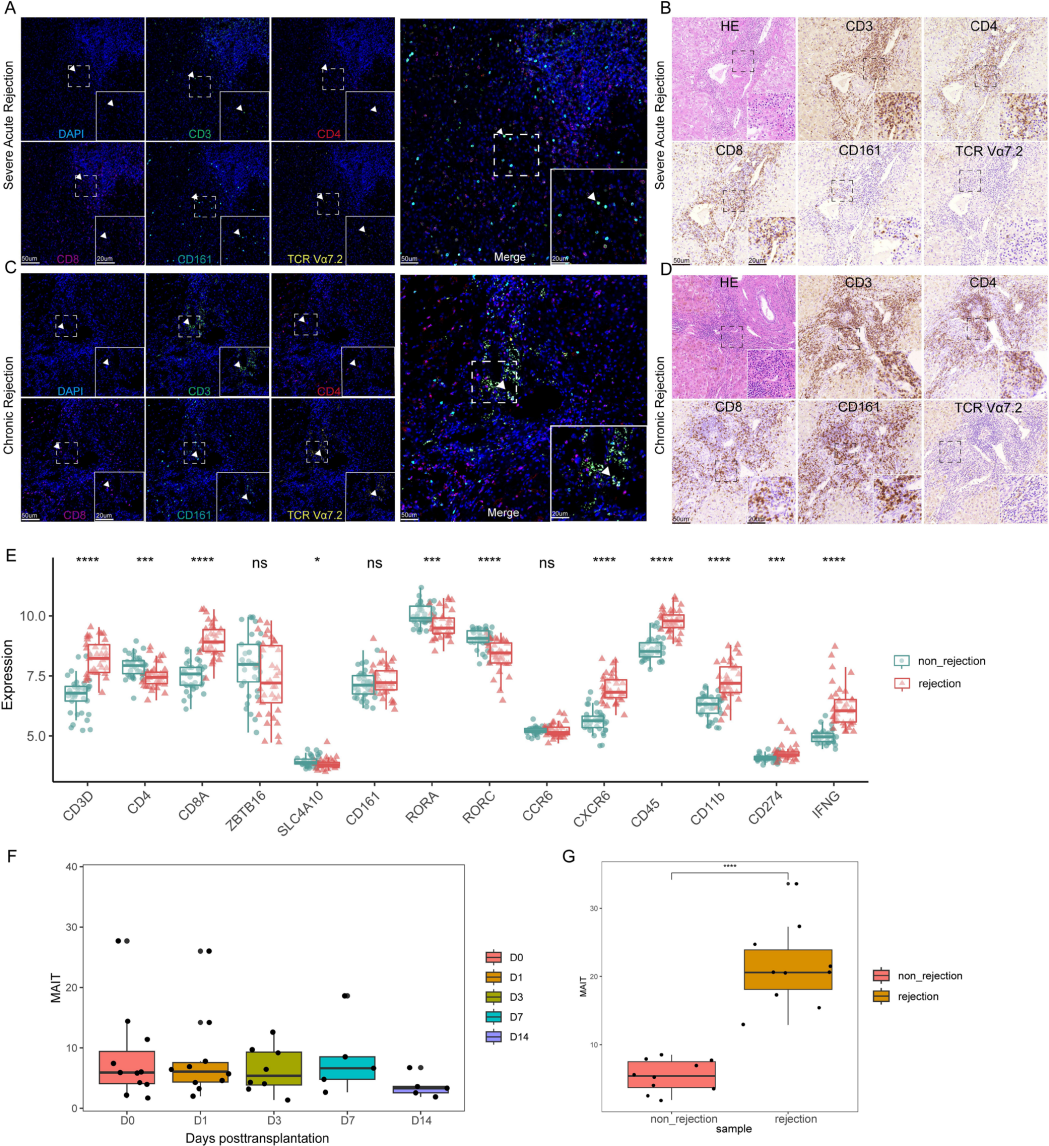
Spatial localisation and clinical validation of MAIT cells in liver allograft rejection. (A, C) Multiplex immunohistochemical (mIHC) analysis of severe acute rejection (sAR) and chronic rejection (CR) tissues. Staining: DAPI (blue), CD3 (green), CD4 (red), CD8 (pink), CD161 (cyan), and TCR Vα7.2 (yellow). Arrows indicate CD8 + CD161 + TCR Vα7.2+ MAIT cells. Scale bars: 50 μm (main), 20 μm (insets). (B, D) Representative H&E and IHC staining (CD3, CD4, CD8, CD161, TCR Vα7.2) in CR, mAR, and sAR samples. Note the perivascular accumulation of MAIT cells. Scale bars: 100 μm (main), 20 μm (insets). (E) Differential expression of MAIT‐associated genes in non‐rejection (*n* = 37) vs. rejection (*n* = 37) cohorts (Bulk RNA‐seq). (F) Longitudinal changes of circulating MR1‐tetramer+ MAIT cells in liver transplant recipients at indicated post‐operative time points (D0 = 11, D1 = 10, D3 = 8, D7 = 5, D14 = 6). (G) Frequency of peripheral MR1‐tetramer+ MAIT cells in stable recipients compared to those with biopsy‐proven rejection (*n* = 10) and non‐rejection (*n* = 10).

Using TCR Vα7.2 and CD161, we identified MAIT cells in human liver tissues under chronic rejection (CR), mild acute rejection (mAR), and severe acute rejection (sAR). Spatially, we observed distinct distribution patterns between rejection types. In acute rejection (AR), MAIT cells were primarily recruited to portal tracts with moderate perivascular infiltration (Figures 2A,C and S2). In contrast, CR tissues exhibited a significantly denser accumulation of MAIT cells specifically localised around blood vessels and fibrotic septa (Figure 2B,D). This histological gradient was fully reconciled with our bulk RNA‐seq analysis, which showed a robust upregulation of MAIT‐associated transcripts (KLRB1, SLC4A10) in rejection samples, confirming that the spatial enrichment reflects a genuine expansion of the MAIT cell compartment.

However, ZBTB16 and SLC4A10 expression conflicted with prior conclusions, indicating that not all MAIT markers displayed significant RNA‐level differences between rejection and non‐rejection states (Figure 2E).

Finally, we performed longitudinal monitoring of circulating MAIT cells using MR1‐tetramer staining. We observed dynamic changes in peripheral MAIT cell frequencies during the post‐operative period (Figures 2F and S2). Crucially, patients with rejection exhibited significantly higher frequencies of circulating MR1‐tetramer+ MAIT cells compared to stable recipients (Figure 2G), underscoring the clinical relevance of MAIT cells as potential biomarkers for rejection.

### 2.3 Establishment of Murine LT Model and Evaluation of Rejection Status

**FIGURE 3:**
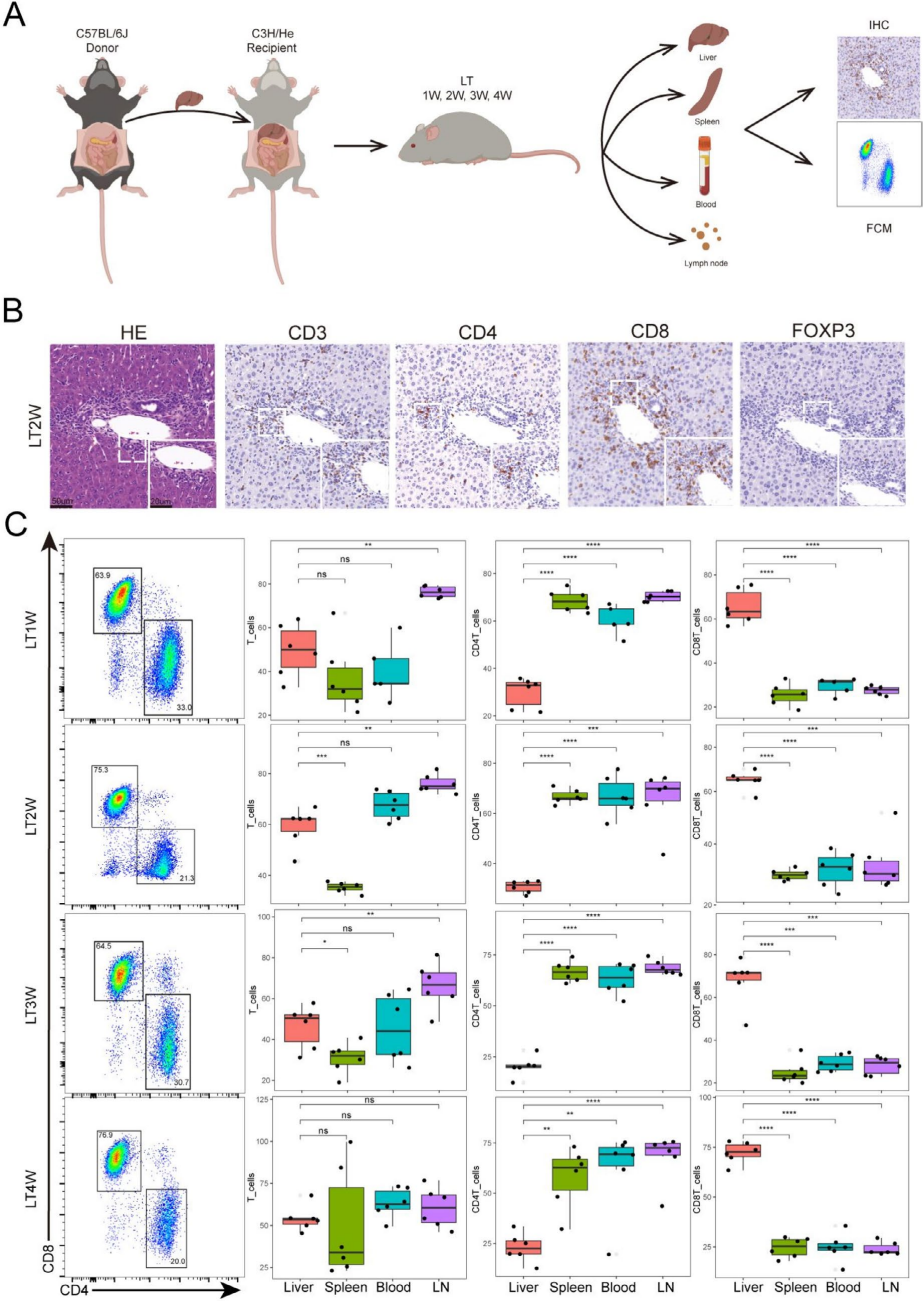
Establishment and immune characterisation of the murine liver transplantation (LT) model. (A) Schematic of the orthotopic liver transplantation model (C57BL/6 or C3H donors/recipients). Samples were collected at 1, 2, 3, and 4 weeks post‐transplantation (LT1W–LT4W). (B) Representative H&E and IHC staining (CD3, CD4, CD8, FOXP3) of liver allografts at LT2W. Scale bars: 50 μm (main), 20 μm (insets). (C) Temporal distribution of total T, CD4+ T, and CD8+ T cells in the liver, spleen, blood, and lymph nodes (LN) post‐transplantation.

To further explore the role of MAIT cells in the rejection reaction after liver transplantation, we established a series of mouse LT models at different time points, specifically at post‐transplantation 1 week (LT1W), 2 weeks (LT2W), 3 weeks (LT3W), and 4 weeks (LT4W). Samples from the liver, spleen, blood, and lymph nodes were collected for flow cytometry analysis (Figure 3A).

HE staining revealed significant inflammatory cell infiltration and vascular endothelial shedding, particularly at LT1W and LT2W post‐transplantation. IHC analysis of CD3, CD4, and CD8 demonstrated robust T cell infiltration after liver transplantation, with CD8 + T cells predominating, highlighting their central role in post‐transplant rejection. FOXP3 expression, indicative of regulatory T cells, was sparse across LT1W to LT4W. These findings strongly suggest the presence of rejection responses, with the most severe rejection occurring at LT2W, followed by a gradual decline in severity by LT3W (Figures 3B and S3).

After LT, the proportion of T cells was redistributed in various tissues (liver, spleen, peripheral blood, LN). At each time point after liver transplantation, the infiltration of T cells in various tissues was significantly higher than that in normal mice (Figures 3C and S5). Amongst them, the proportion of CD4 + T cells gradually decreased with time, whilst the proportion of CD8 + T cells showed a gradual increasing trend (Figure S5). The infiltration in the liver was particularly obvious, indicating that the liver was the first to be involved after liver transplantation. However, the changes in T cells and their subsets showed a single trend, and we cannot observe whether there was a tendency of immune tolerance in the transplanted mice.

### 2.4 Dynamic Changes of CD8 + MAIT Cells in Murine LT Model

**FIGURE 4:**
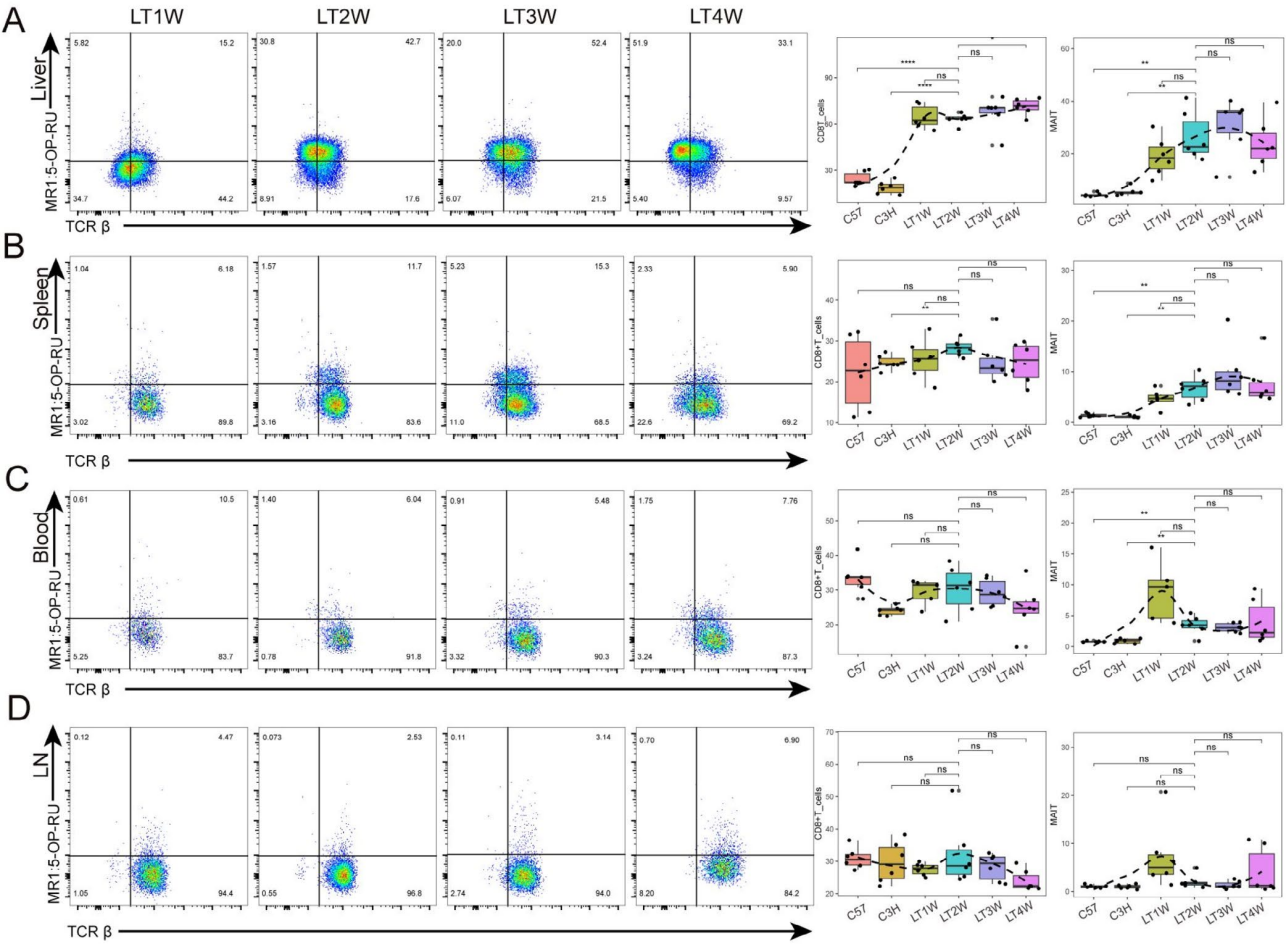
Temporal dynamics of CD8+ T and MAIT cells post‐transplantation. Quantification of CD8+ T cell and CD8+ MAIT cell frequencies across the liver, spleen, blood, and LN from LT1W to LT4W. Data illustrate the dynamic expansion of cytotoxic subsets during rejection.

To further explore the dynamics and tissue distribution of MAIT after liver transplantation, we demonstrated the flow cytometry using CD3, CD4, CD8, TCRβ, MR1 tetramer (5‐OP‐RU to detect MAIT cells and 6‐FP for negative control) and checkpoint PD1 (Figure S3). Then, we focus on CD8 + MAIT, detected by TCRβ and MR1 tetramer (5‐OP‐RU). The T cells, CD8 + T cells and CD8 + MAIT significantly increased after liver transplantation in liver, spleen, blood and lymph node. The CD4 + T cells declined or had no noticeable changes after liver transplantation (Figure S4). In the liver and spleen, CD8 + MAIT demonstrated a clear trend, rising in LT1W and LT2W, peaking in LT3W, and declining in LT4W (Figure 4A,B). This trend is different from our results of HE and IHC, we suggest that MAIT plays a delayed role in LT rejection. There is no temporal difference in CD8 + MAIT occupancy in blood and lymph nodes indicating that the remodelling of the immune microenvironment by MAIT cells is restricted to the liver and spleen (Figure 4C,D).

We also define the CD4 + MAIT using the same strategy. The trend of CD4 + MAIT is broadly similar to the CD8 + MAIT, but the proportion in T cells is far less than CD8 + MAIT (Figure S5). Thus, we thought CD8 + MAIT still constitutes a dominant position in liver transplantation rejection.

### 2.5 MAIT Cells Are a Potential Target of PD1/PDL1 Axis in Murine Liver Transplantation

**FIGURE 5:**
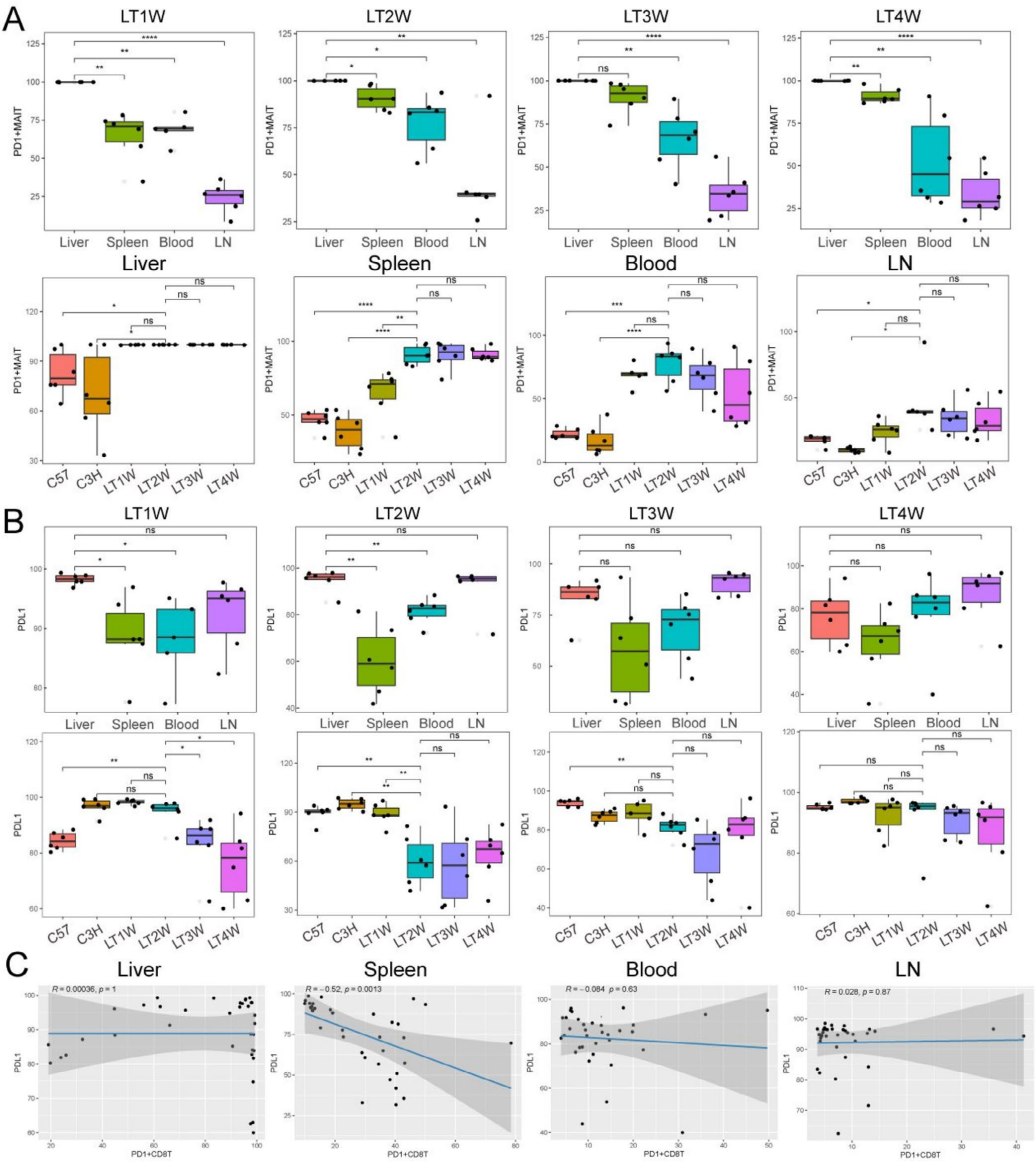
Correlation of the PD1/PDL1 axis between MAIT and myeloid cells. (A, B) Longitudinal expression kinetics of PD1 on CD8+ MAIT cells (A) and PDL1 on myeloid cells (B) across tissues and time points. (C) Correlation analysis between the frequency of PD1+ CD8+ MAIT cells and PDL1+ myeloid cells.

In our investigation of CD8 + MAIT cells, we focused on the programmed cell death protein 1 (PD1) subset. The expression of PD1 in the liver was notably high in both normal and transplanted mice, approaching 100% in the transplanted group (Figure 5A). This suggests a low immune status, which may predispose to rejection. The percentage of PD1 + MAIT cells in lymph nodes was generally lower compared to the other three tissues examined. Subsequently, we assessed the proportions of PD1 + CD8 and PDL1 in myeloid cells (Figure S6). The results indicated that PDL1 levels were also elevated in the liver, surpassing those of PD1 in the lymph nodes (Figure 5B). There are several differences in the expression of PD1 between CD8 + T cells and MAIT cells, highlighting that CD8 + MAIT cells predominantly engage in the PD1/PDL1 axis for immune function. We conducted a correlation analysis of the distribution of PD1 and PDL1 across various tissues, revealing a close proportional relationship that resulted in a linear correlation. Although PD1 and PDL1 are consistently highly expressed in the liver, correlation analysis shows that there is no statistically significant linear correlation between PD1 + CD8 + T cells and PDL1 in the liver. Similarly, there is no correlation in peripheral blood and lymph nodes. However, in the spleen, a negative correlation is observed (Figure 5C).

### 2.6 MR1KO Murine Liver Transplantation Model Attenuates Allograft Rejection

**FIGURE 6:**
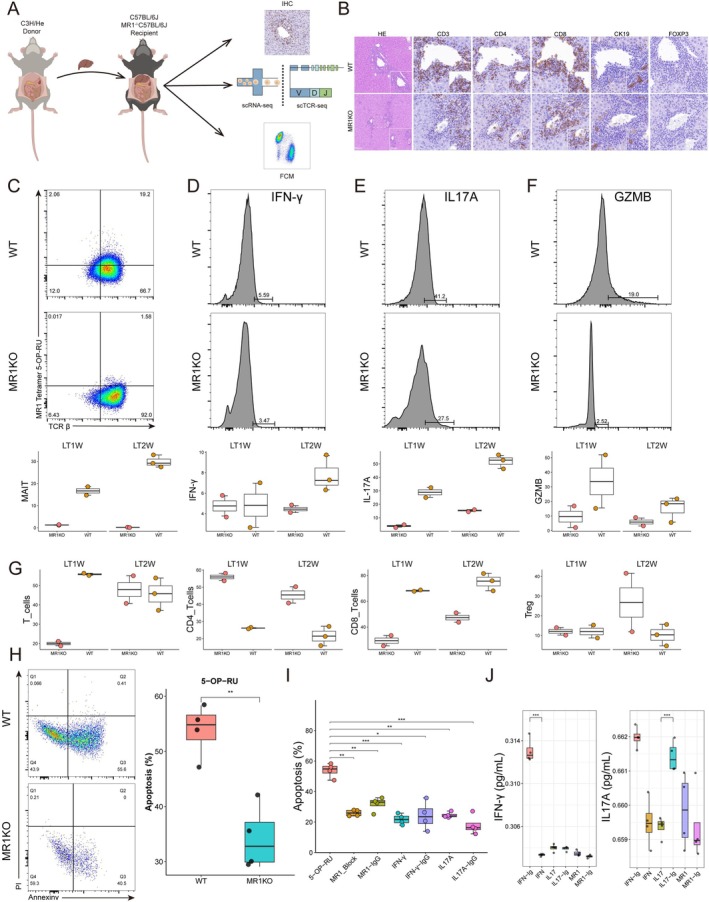
MR1 deficiency attenuates liver allograft rejection. (A) Experimental design comparing Wild‐Type (WT) and Mr1KO recipients (*n* = 4). (B) Representative H&E and IHC staining (CD3, CD4, CD8, CK19, FOXP3) of liver allografts at LT1W. Scale bars: 50 μm (main), 20 μm (insets). (C–F) Flow cytometry quantification of total MAIT cells (C) and intracellular cytokines: IFN‐γ (D), IL‐17A (E), and Granzyme B (F). (G) Proportions of T cell subsets (Total, CD4+, CD8+, Treg) in WT versus Mr1KO recipients. (H) Representative plots of the in vitro co‐culture blockade assay involving hepatic MAIT cells and primary hepatocytes. (I–J) Functional assessment of MAIT cell cytotoxicity upon cytokine blockade.

To elucidate the role of MAIT cells in liver transplant rejection, we established mouse models using C3H donors and either wild‐type (WT) or MR1KO (knockout) C57BL/6 recipients. Liver, spleen, blood, and lymph nodes were collected at LT1W and LT2W for flow cytometry and HE assessment (Figure 6A). HE staining showed more severe portal inflammation in WT controls, and IHC revealed stronger CD3 + T cell infiltration and CD8 + cytotoxic accumulation—hallmarks of rejection—whereas MR1KO mice had higher CD4 + T cell levels. FOXP3 + Treg density and CK19 staining were comparable between groups (Figures 6B and S7). Flow cytometry confirmed MAIT cell absence in MR1KO recipients (Figure 6C). WT recipients exhibited conserved CD8 + MAIT dynamics, with PD1hi trajectories shared across strains (Figures 4 and S7). Multi‐organ analysis indicated systemic MAIT redistribution (Figure S8).

Cytokine profiling revealed distinct MAIT subset behaviours: MAIT17‐associated IL‐17A was persistently suppressed, whilst MAIT1‐related IFN‐γ only transiently decreased at LT2W, suggesting MAIT17 involvement in acute rejection (Figure 6D,E). GZMB was consistently downregulated in MR1KO mice across all tissues, indicating systemic impairment of cytotoxic responses (Figures 6F and S9).

Flow cytometry revealed immunophenotypic divergence: MR1KO recipients had reduced T cell infiltration at LT1W, with preferential CD8+ loss and CD4+ accumulation (Figure 6G), correlating with milder rejection. Despite overall attenuation, CD8 + T cells remained hyperactivated in MR1KO livers, although systemic depletion occurred in spleen, blood, and lymph nodes (Figure S10).

Treg cells expanded in WT livers post‐transplant, with elevated PD1 and TIGIT, suggesting enhanced local immunosuppression (Figure S11). MR1KO mice showed superior Treg expansion, exceeding WT levels by LT2W in liver and spleen, with no significant changes in blood, lymph nodes and thymus (Figures 6G, S11, and S12). Whilst Treg increased systemically, checkpoint upregulation was liver‐specific, indicating that MAIT cell depletion promotes global tolerance mainly through Treg expansion, with localised checkpoint modulation within grafts.

To further delineate the specific effector mechanisms of MAIT cells, we conducted in vitro co‐culture experiments utilising primary hepatocytes as target cells and purified hepatic MAIT cells as effectors (Figure 6H). The cytotoxic effect of MAIT cells was significantly abrogated by the addition of neutralising antibodies targeting IL‐17A or IFN‐γ (Figure 6I). Quantitative analysis confirmed that blockade of either cytokine, particularly IL‐17A, resulted in a marked reduction in hepatocyte apoptosis/injury (Figures 6J and S13). These data provide direct evidence that MAIT cells exert their pathological effects in liver allografts through the secretion of proinflammatory cytokines.

### 2.7 Single‐Cell Transcriptional Profiling Uncovered Immune Subset Remodelling in MR1KO LT Recipients

**FIGURE 7:**
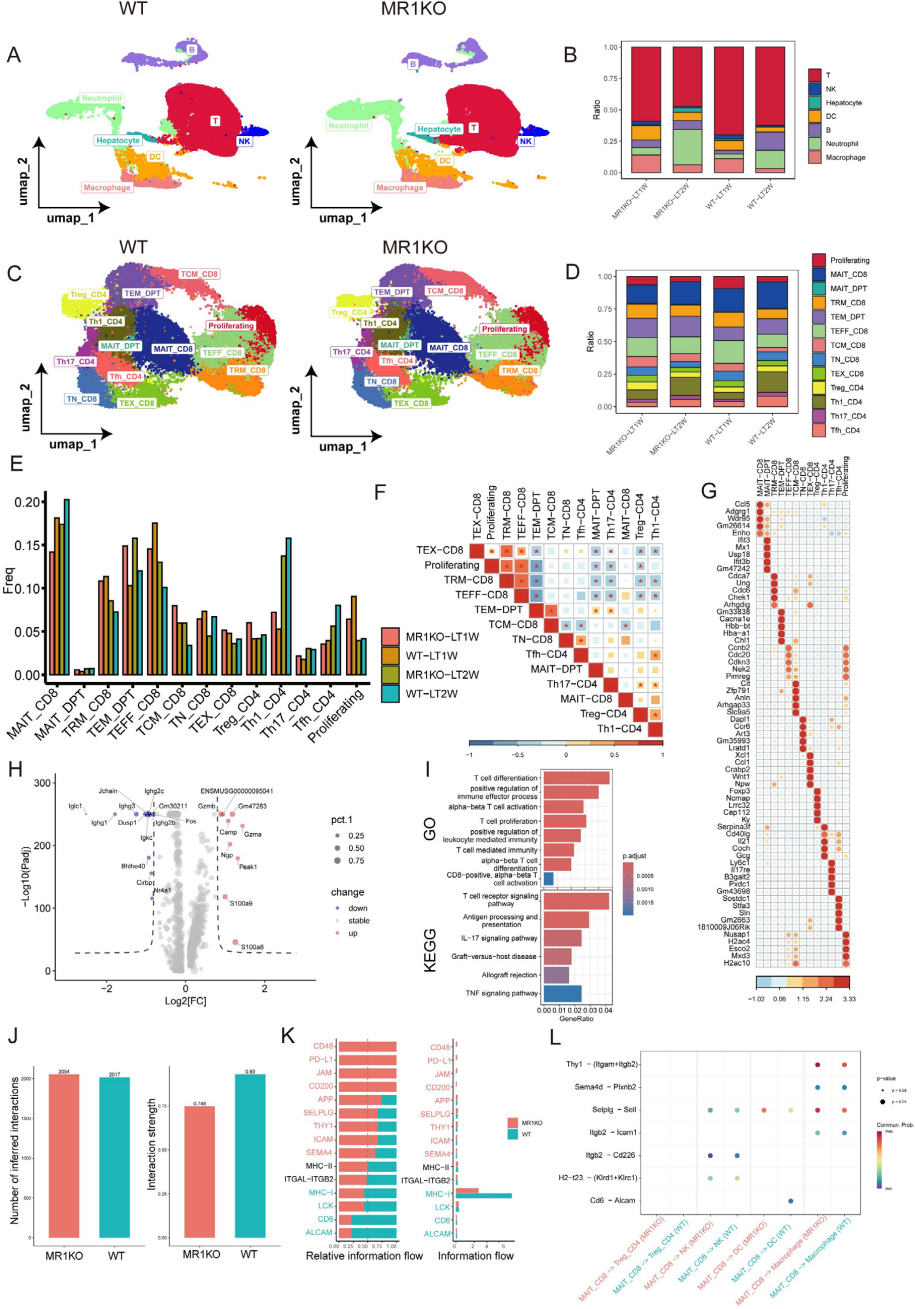
Single‐cell immune profiling of MR1‐deficient allografts. (A, B) UMAP visualisation of immune cell lineages (A) and their relative proportions (B) in WT and Mr1KO grafts. (C–E) Clustering (C), distribution (D), and composition (E) of T cell subsets. (F–G) Correlation heatmap of gene expression (F) and top marker genes for each cluster (G). (H, I) Volcano plot of DEGs (H) and corresponding GO/KEGG pathway enrichment (I) in Mr1KO vs. WT groups. (J–L) CellChat analysis comparing interaction number (J), strength (K), and signalling pathways (L).

To delineate MAIT mediated immunoregulation in transplantation, we performed single‐cell RNA sequencing of hepatic allografts (*n* = 110,821; Figure S14) and annotated seven major lineages: T, B, dendritic, NK, hepatocyte, neutrophil, and macrophage cells (Figure S14). Integrated UMAP visualisation and differential expression analysis revealed distinct transcriptomic landscapes between WT and MR1KO recipients (Figure 7A,B). T cell frequencies were reduced in MR1KO grafts, suggesting attenuated rejection.

From 64,986 T cells, we identified 21 transcriptionally distinct subsets, including MAIT, TRM, Treg, TEX, memory, naïve, effector, helper, and proliferating populations (Figure S14). UMAP visualisation revealed genotype‐specific T cell state distributions (Figure 7C). MAIT cells were the predominant subset in WT recipients, whereas MR1KO LT1W grafts showed expanded Treg infiltration (Figure 7D,E).

Correlation heatmap analysis indicated a significant inverse relationship between MAIT and TCM cells, suggesting divergent functional polarisation (Figure 7F, Table S4). MAIT cells overexpressed immunomodulatory factors, including Ccl5, Adgrg1, Wdr95, Gm26614, and Enho (Figure 7G). Differential expression analysis identified 1031 significant genes, with 22 showing consistent directional change (0.62% validation rate; Figure 7H, Table S5). Functional enrichment revealed pathways related to T cell activation, IL‐17 signalling, and allograft rejection, indicating that MR1 deficiency mitigates rejection via immunomodulation of T cell effector and antigen presentation networks (Figure 7I, Table S6).

Ligand–receptor analysis showed comparable interaction numbers but stronger signalling probability in WT recipients, indicating heightened alloreactive synapse activity (Figure 7J). MHC‐I–mediated interactions dominated WT microenvironments, whilst MR1KO grafts retained minor checkpoint signalling (e.g., CD48, PDL1; Figure 7K). Although some pathways were shared, MR1 deficiency led to altered interaction strength and significance, particularly with Treg, NK, and macrophage subsets, suggesting functional reprogramming of MAIT regulatory networks (Figures 7L and S14, Table S7). Furthermore, we performed the lineage relationships amongst CD8 + MAIT subsets, which were clustered into six clusters and annotated as MAIT, MAIT1 and MAIT17 (Figure S15), using CytoTRACE to predict the differentiation potential (Figure S15). We got the same results with the human CD8 + MAIT profiles, which further validate this differentiation of MAIT cells in liver transplant microenvironment.

### 2.8 TCR Repertoire Alteration Revealing MR1KO Mitigates Rejection in LT Murine

**FIGURE 8:**
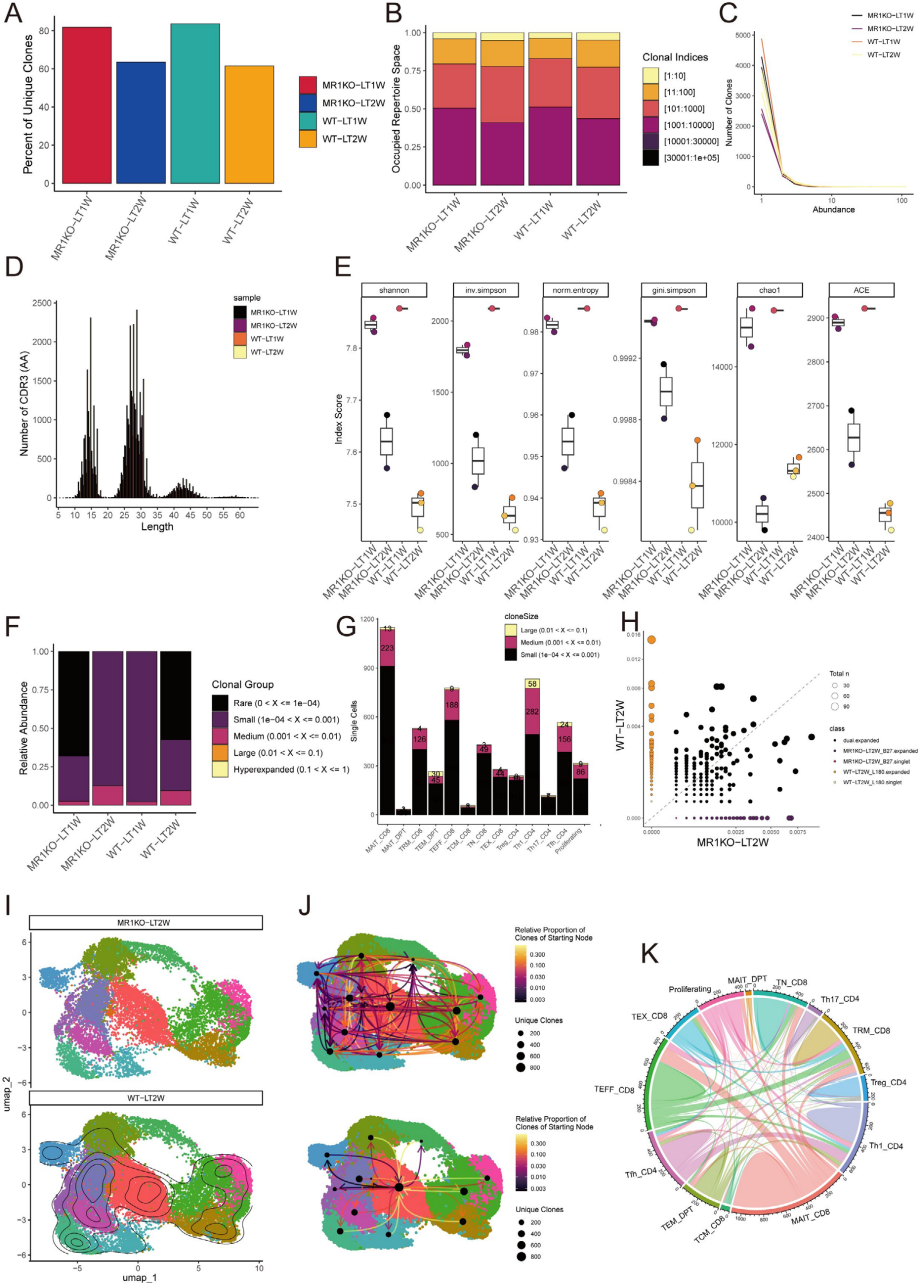
MR1‐dependent reshaping of the TCR repertoire. (A) Percentage of unique TCRβ clones in WT vs. Mr1KO groups. (B, C) Clonal space occupancy (B) and rank‐abundance curves (C) illustrating clonal expansion. (D) Distribution of CDR3β amino acid lengths. (E) TCR diversity indices (Shannon, Inverse Simpson, Gini‐Simpson, Chao1, ACE). (F) Relative abundance of clones categorised by frequency (Rare to Hyperexpanded). (G) Clonal expansion analysis in MAIT vs. non‐MAIT T cells. (H) Clonal overlap scatter plot between WT and Mr1KO groups at LT2W. (I, J) UMAP visualisation of TCR clonotypes (I) and clonal trajectory analysis (J). (K) Chord diagram showing clonotype sharing across T cell subsets.

To investigate how MAIT cell clonal architecture influences liver transplant rejection, we performed single‐cell TCR sequencing in WT and MR1KO mice. MR1KO mice exhibited a significantly higher proportion of unique TCR clones than WT, especially at LT2W, suggesting restricted clonal expansion in the absence of MR1 (Figures 8A and S16). Repertoire space analysis further showed that MR1KO repertoires were skewed towards low‐frequency clones ([1:100], [101:1000]), whereas WT‐LT2W was dominated by larger clonotypes ([1001:30000]) (Figure 8B). Clone abundance curves confirmed more high‐frequency clones and reduced diversity in WT, consistent with a stronger rejection response (Figure 8C).

Heatmap and phylogenetic analyses revealed distinct segregation of TCR usage between MR1KO and WT mice, indicating that MR1 signalling shapes immunodominance hierarchies and alloreactive T cell repertoires (Figure S16). Several TCR clones were uniquely enriched in each group, suggesting distinct drivers of rejection (Figure S16).

CDR3 length distributions were similar across groups, with conserved peaks around 15–18 and 25–30 amino acids, reflecting the invariant nature of MAIT TCRβ chains (Figure 8D). However, diversity indices revealed pronounced reductions in Shannon, inverse Simpson, normalised entropy, and Chao1/ACE in WT‐LT2W, whereas MR1KO LT1W maintained the highest clonal richness and evenness (Figure 8E), indicating that post‐transplant MAIT clonal expansion is MR1‐dependent.

Clonal group distribution showed predominance of rare and small clones in MR1KO mice, whereas WT‐LT2W contained more medium and large clones (Figure 8F,G). Cell‐type‐specific analysis confirmed expanded clones in WT‐LT2W MAIT cells, whilst MR1KO MAIT cells remained largely unexpanded (Figures 8H and S16). Clone‐sharing analysis further revealed minimal overlap between MR1KO and WT at LT2W, underscoring MR1‐dependent divergence in clonal trajectories (Figures 8I and S15).

Single‐cell TCR profiling showed that MR1KO mice failed to form large density hotspots towards effector subsets (e.g., TEFF, TRM, TEX), indicating a specific role for MAIT cells in promoting T cell clonal expansion and migration (Figure 8I). Extensive TCR clonal sharing was observed amongst T cell subsets—particularly MAIT, TEFF, proliferating, and Th1 cells—suggesting that MAIT activation enhances effector responses and amplifies acute graft rejection (Figure 8J,K).

## 3 Discussion

This study for the first time delineated the dynamic changes of MAIT cells in a murine liver transplantation model, uncovering the pivotal role of MAIT cells in liver transplantation rejection. The alleviation of LT rejection upon MAIT cell deficiency due to MR1 knockout was further validated using MR1KO murine liver transplantation model. Additionally, at the single‐cell level, the mechanism underlying this alleviation was explored through the analysis of TCR clonotype changes during the remodelling of the transplant immune microenvironment. We found that in the immune microenvironment of liver transplantation, MAIT cells promote the occurrence of post‐transplant rejection. After transplantation, MAIT cells are rapidly activated through MR1 to exert their effector functions, releasing factors such as IFN‐γ, IL‐17A, and GZMB, which exacerbate tissue damage and disrupt the homeostasis of memory T cells. Meanwhile, MAIT cells play a critical role in the remodelling of the TCR repertoire after transplantation. MR1‐mediated MAIT cells promote obvious clonal expansion towards a single dominant clone and reduce clonal diversity, thereby intensifying the rejection response.

Previous studies have investigated the relationship between MAIT cells and immunosuppressants in the peripheral blood of liver and kidney transplant patients [26], but have not revealed the role of MAIT cells in liver transplantation rejection. A recent study has also highlighted the presence of MAIT cells in the context of liver allografts [27]. Whilst that study provided valuable insights into the MAIT cells in early transplanted liver, it still focuses more on liver failure, our work significantly advances the field by dissecting the dynamics and clonal architecture of MAIT cells with higher resolution. Unlike previous studies that primarily relied on correlative data, our use of the MR1KO orthotopic liver transplantation model establishes a direct causal link between MAIT cell activation and rejection outcomes. Furthermore, we uniquely employed single‐cell TCR sequencing to reveal how MAIT cells influence the broader T‐cell repertoire remodelling, providing a mechanistic explanation that extends beyond simple abundance changes.

Subsequently, we further explored the plasticity of MAIT cells in liver transplantation using a murine liver transplantation model. Our findings underscore the potent immunomodulatory function of MAIT cells, which acts as a bridge between innate sensing and adaptive effector responses. During the remodelling of the immune microenvironment after liver transplantation, stimulation from different donor antigens activates various immune cells, leading to rejection [28, 29, 30]. Due to the unique characteristics of MAIT cells, they exhibit dual activation properties. Early activation occurs directly via the MR1 molecule, triggering their effector functions and mediating rejection. Sustained activation of MR1 leads to continuous expansion of MAIT cells during the early rejection phase (LT1W–LT3W) in liver‐transplanted mice. Notably, C3H mice show better tolerance [31], and immune tolerance gradually emerges at LT4W, followed by a decline in MAIT cell frequency. Therefore, the MR1‐MAIT axis could serve as a marker for monitoring post‐liver‐transplantation immune status. In the transplanted liver, CD8 + MAIT cells highly express PD1. The high expression of immune checkpoints can lead to immune escape [32], thereby predisposing to the occurrence of rejection. Nearly all MAIT cells surface‐express PD1 [4, 33], indicating that CD8 + MAIT cells could serve as potential therapeutic targets for PD1/PDL1 blockade.

By leveraging an orthotopic liver transplantation model in MR1‐deficient mice integrated with single‐cell TCR tracking, our study not only establishes a causal link identifying MAIT cells as key drivers of rejection but, more importantly, unveils their profound immunomodulatory function as a critical bridge between innate and adaptive immunity. However, due to the unique development of MAIT cells, which is tightly linked to MR1, knocking out MR1 differs from knocking out other gene phenotypes: it directly inhibits MAIT cell generation and development, leading to global MAIT cell deficiency in mice [34]. This approach more effectively verifies the role of MAIT cells in transplant rejection. HE and IHC staining evaluation showed that MR1KO effectively alleviated the rejection status in LT mice. We observed changes in the immune status after MR1 knockout through multiple approaches and revealed its mechanisms. After MR1 knockout, the infiltration of T cells and CD8 + T cells in the liver was significantly reduced, and the proportion of cytotoxic substances such as IFN‐γ, IL‐17A, and GZMB in various tissues was decreased, which effectively mitigated the occurrence of rejection [35, 36, 37]. Furthermore, through crosstalk with myeloid lineages, specifically macrophages and dendritic cells, activated MAIT cells to antigen‐presenting cells, thereby amplifying the downstream adaptive immune cascade. This core regulatory role is mechanistically deconstructed in the tolerance phenotype observed in MR1KO recipients, necessitating a distinction between primary and secondary effects: the absence of MR1 primarily results in the direct ablation of MAIT cells and the abrogation of the early cytotoxic and cytokine storm, effectively removing the initiating trigger of acute injury. Consequently, the relief from this primary insult triggers a secondary remodelling of the immune microenvironment, characterised by the compensatory expansion of Treg [38, 39], liberated from MAIT cell‐mediated suppression and the polarisation of macrophages towards an anti‐inflammatory phenotype. Thus, these concerted primary deficits and secondary protective mechanisms collectively contribute to the significant mitigation of allograft rejection [40, 41].

The changes in the TCR repertoire within the immune microenvironment after liver transplantation are of great significance for predicting rejection. A significant decrease in the number of TCR clones, along with the rapid expansion of specific monoclonal clones, often indicates the occurrence of rejection, whilst maintaining high TCR clonal diversity is typically associated with immune tolerance [42, 43, 44]. Our research data show that after MR1 knockout, transplanted mice maintain a more diverse immune response, whilst the WT group exhibits typical immune system stimulation leading to massive expansion of specific T cells. Compared with WT mice, MR1KO mice are more likely to achieve immune tolerance. Due to the unique characteristics of MAIT cells, the clonal expansion of their TCRβ chains can reflect antigen‐specific immune responses or disease states, offering high traceability for dynamic monitoring [45, 46]. Through TCR analysis, our study revealed that the MR1‐MAIT axis orchestrates the remodelling of the TCR clonal repertoire, leading to reduced TCR clonotype diversity and the dominance of single or oligoclonal expansions, which in turn triggers transplant rejection. Notably, during the rejection process, MAIT cells do not act in isolation but form interconnected networks with classical CD8+ cytotoxic T cells and Th cells, collectively driving the rejection cascade.

The limitations of our study include the lack of exploration into the functional roles of transcription factors in MAIT cells during LT, as well as the developmental and differentiation trajectories of MAIT cells within the transplant immune microenvironment. Additionally, it remains unclear whether distinct regulatory mechanisms dominate MAIT cell activation in acute versus chronic rejection.

In summary, our study reveals the critical role of MAIT cells in LT rejection and the key mechanism by inducing rejection through influencing TCR repertoire remodelling, whilst also mapping their dynamic changes during the rejection process. In the future, it may be feasible to monitor the incidence of rejection in clinical patients by assessing the frequency of MAIT cell changes and MR1 activity, and to regulate rejection by modulating the MR1‐MAIT axis, thereby providing novel strategies for preventing and ameliorating rejection after liver transplantation.

## Author Contributions

B.W., H.C., and X.L. contributed to the research design. H.C., X.L., X.Z., X.W., Z.J., R.D., and Y.L. performed basic experiments. H.C, X.L., Y.W., S.L., W.S., D.W., D.T., K.Z., B.W., and J.C. contributed to the data management and statistical analysis. B.W., H.C. and X.L. wrote the manuscript. All authors contributed to the article and approved the submitted version.

## Funding

This work was supported by Science Foundation of Fujian Medical University Union Hospital (No. 2020XH009 and 2020XH012), National Natural Science Foundation of China (Grant No. 82470686), Scientific and technological project of Henan Province (Grant No. 242102310072) and Fujian Provincial Natural Science Foundation of China (No. 2022J01738).

## Disclosure

Male C57BL/6J mice and male C3H/He mice are donors and male C3H/He mice are recipients. All animals were 8–10 weeks old (body weight = 23 ± 2 g), purchased from Charles River, Beijing (www.vitalriver.com) and kept in Qingdao University (Issue No. 20240510C57C3H9020241210134) in a specific pathogen free (SPF) environment. MR1KO mice (Cat. No. NM‐KO‐190119) were purchased from Shanghai Model Organisms Center Inc. Orthotopic liver transplantation (OLT) was performed under isoflurane inhalation anaesthesia according to established procedures [47]. Syngeneic OLT (Non‐Rejection Control): C57BL/6 mice served as both donors and recipients. These grafts are genetically identical and result in long‐term survival without immunosuppression. Allogeneic OLT (Acute Rejection): Livers from C57BL/6 donors were transplanted into C3H recipients (or C3H to MR1KO C57BL/6). This fully MHC‐mismatched combination results in severe acute rejection.

## Ethics Statement

The study protocol was approved by the Ethics Committee of the Affiliated Hospital of Qingdao University (IRB number: QYFYWZLL29376). Verbal informed consent was obtained from all patients prior to their inclusion in the study. This study encompassed liver biopsies of patients who underwent liver transplantation (LT) at the Organ Transplantation Center, the Affiliated Hospital of Qingdao University.

## Conflicts of Interest

The authors declare no conflicts of interest.

## Supporting information


**Figure S1:** Interaction and signature analysis of MAIT cells in human liver allografts. (A) Dot plot illustrating cellular interactions between CD8+ MAIT cells and myeloid subsets in non‐rejection (NR) vs. rejection (R) samples. (B) Violin plots showing MAIT1 signature scores across four clusters.

**Figure S2:** Multiplex immunohistochemical (mIHC) analysis of MAIT cells. Representative mIHC images of mild acute rejection (mAR) and chronic rejection (CR) liver tissues. Staining: DAPI (blue), CD3 (green), CD4 (red), CD8 (pink), CD161 (cyan), and TCR Vα7.2 (yellow). Arrows indicate CD8 + CD161 + TCR Vα7.2+ MAIT cells. Scale bars: 50 μm (main), 20 μm (insets).

**Figure S3:** Histological assessment and flow cytometry gating strategy. (A) H&E and IHC staining (CD3, CD4, CD8, FOXP3) of liver tissues at 1, 3, and 4 weeks post‐transplantation (LT1W–LT4W). (B) Flow cytometry gating strategy for identifying CD8+ MAIT cells.

**Figure S4:** T cell profiling in naïve mice. Flow cytometry analysis of T cell subsets in naïve C57BL/6 and C3H mice.

**Figure S5:** Longitudinal dynamics of T cells and MAIT cells in liver transplantation. (A) Proportions of total T and CD4+ T cells in liver allografts from week 1 to 4 (LT1W–LT4W). (B‐C) Distribution of CD8+ (B) and CD4+ (C) MAIT cells across indicated tissues in control (C57, C3H) and transplant groups over time.

**Figure S6:** PD‐1 expression kinetics and myeloid gating strategy. (A) Frequency of PD‐1 + CD8+ T cells across tissues at different post‐transplant time points. (B) Flow cytometry gating strategy for PD‐L1+ myeloid cells.

**Figure S7:** Impact of MR1 deficiency on hepatic T cell subsets. (A) Representative H&E and IHC staining (CD3, CD4, CD8, CK19, FOXP3) of liver allografts at 2 weeks post‐transplant (LT2W). Scale bars: 50 μm (main), 20 μm (insets). (B‐C) Frequencies of CD4+, CD8+, and PD‐1+ MAIT cells in the liver (B) and spleen (C) of WT versus MR1KO recipients over time.

**Figure S8:** Systemic changes in MAIT cell subsets in MR1KO mice. (A‐B) Frequencies of CD4+, CD8+, and PD‐1+ MAIT cells in peripheral blood (A) and lymph nodes (LN) (B) of WT and MR1KO mice. (C) Comparison of CD8+ and PD‐1+ MAIT cell proportions in the thymus (TH).

**Figure S9:** Cytokine and cytotoxicity profiles across tissues. Comparison of IFN‐γ, IL‐17, and Granzyme B (GZMB) expression levels in spleen, blood, lymph nodes, and thymus.

**Figure S10:** Comparative analysis of T cell compartments in WT and MR1KO mice. (A‐B) Proportions of total T, CD4+ T, and CD8+ T cells in the liver, spleen, blood, and lymph nodes (LN) of WT and MR1KO groups.

**Figure S11:** Hepatic and splenic Treg dynamics. (A) Gating strategy for Treg. (B‐C) Temporal changes and comparison of total, PD1+, and TIGIT+ Treg in the liver (B) and spleen (C) of WT and MR1KO mice.

**Figure S12:** Systemic Treg dynamics in lymphoid tissues. (A‐C) Temporal changes and comparison of total, PD1+, and TIGIT+ Treg in the blood (A), lymph nodes (B), and thymus (C) of WT and MR1KO mice.

**Figure S13:** MAIT cell function in intro. (A) Gating strategy for identifying hepatocytes of Annexin V expression. (B) Quantification of cytokine (IFN‐γ, TNF and IL‐17A) and cytotoxic molecule (GZMB) production by co‐culture models in WT and MR1KO.

**Figure S14:** Single‐cell landscape of murine liver allografts. (A‐B) UMAP visualisation of all cell clusters (A) and sample distribution (B‐C) Dot plot of canonical marker genes. (D‐E) UMAP re‐clustering of T cells (D) and sample distribution. (F) Marker genes for T cell subsets. (G) MAIT gene signature scores across clusters. (H) Ligand‐receptor interactions between MAIT cells and macrophages.

**Figure S15:** Single‐cell landscape of MAIT cells in murine liver allografts. (A) UMAP visualisation of CD8 + MAIT cell clusters. (B) VlnPlot shows the Addmoudule Score of MAIT1 and MAIT17. (C) UMAP visualisation of annotated CD8 + MAIT cells. (D) UMAP shows the distribution of CD8 + MAIT cells in samples. (E) UMAP projection and box plot show CD8 + MAIT cells were predicted with differentiation states using CytoTRACE.

**Figure S16:** TCR repertoire analysis. (A) Percentage of unique TCR clones per group. (B) Heatmap of Jaccard similarity coefficients between genotypes. (C) Hierarchical clustering based on clonal characteristics. (D) Clonal expansion proportions within each group. (E) Scatter plot comparing clonal frequencies between MR1KO‐LT1W and WT‐LT1W. (F) Clonal size distribution histograms.

**Table S1:** GO and KEGG pathway enrichment analysis of differentially expressed genes (DEGs) in MAIT cells.

**Table S2:** Intercellular communication analysis between MAIT cells and myeloid cells (Human).

**Table S3:** Differentially expressed genes of MAIT cell subsets.

**Table S4:** Differentially expressed genes across T cell clusters.

**Table S5:** Differentially expressed genes between MR1KO and WT samples.

**Table S6:** GO and KEGG pathway enrichment analysis of DEGs in murine transplant samples.

**Table S7:** Intercellular communication analysis between MAIT cells and myeloid cells.

**Data S1:** Supporting Information.

## Data Availability

The datasets and code supporting the findings of this study are openly accessible in the NGDC Genome Sequence Archive (https://ngdc.cncb.ac.cn/gsa‐human/) under accession numbers HRA002091 and HRA007802 whilst datasets of mouse can be obtained through CRA029807. The computational code can be retrieved from the GitHub repository at https://github.com/Allen272515/sc_data_code, and all processed data and analysis scripts are available from the corresponding author upon reasonable request.
