# Supplementary figures and images for: Single Cell Multi‐Omics Revealing the Important Role of MR1 Mediated MAIT Cells in Maintaining Rejection for Liver Transplantation
Source: Cell Prolif. 2026 Mar 10:e70194. Online ahead of print. doi: 10.1111/cpr.70194 (PMC13325667; doi:10.1111/cpr.70194)

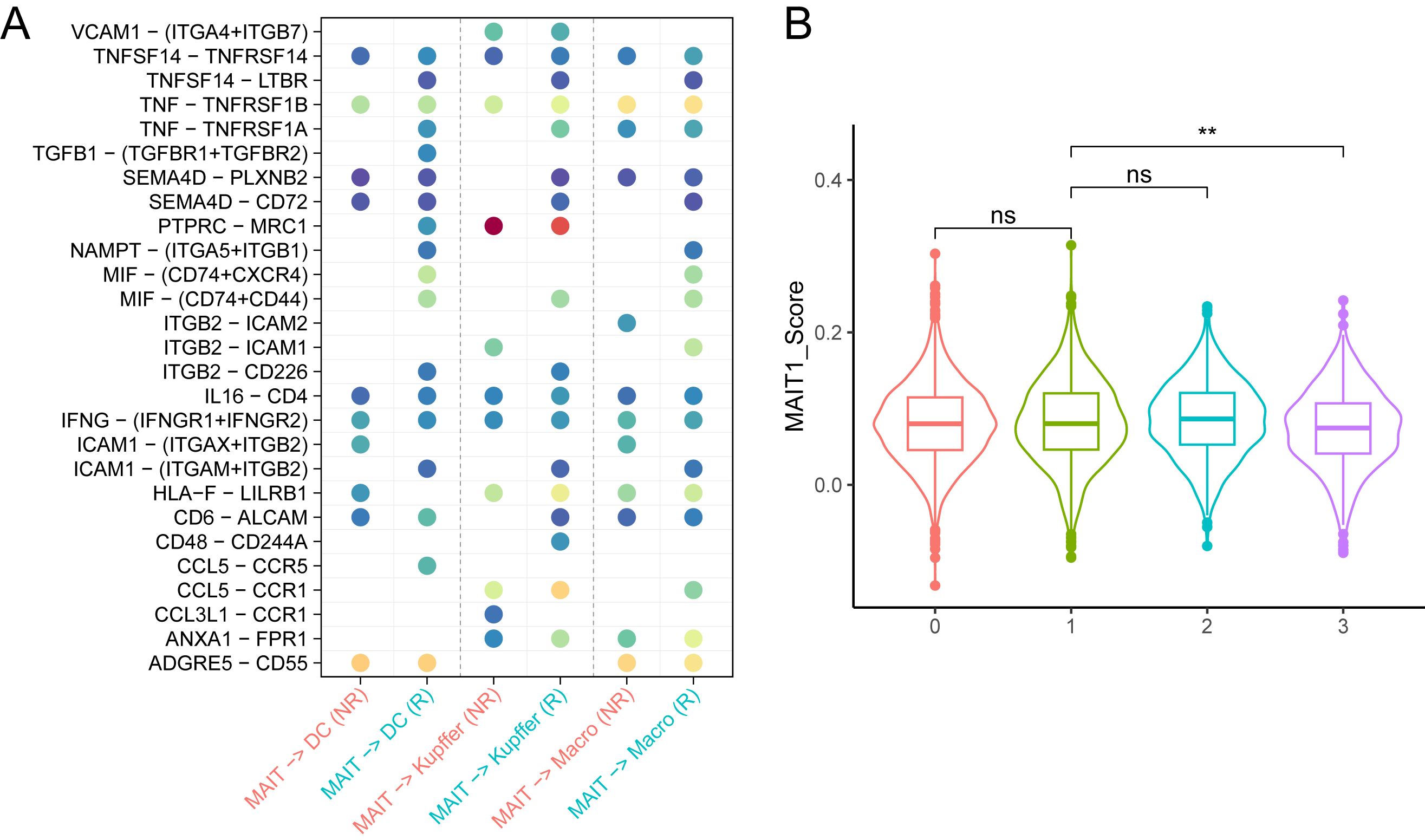

Supplement: Supplementary file 1 — Figure S1: Interaction and signature analysis of MAIT cells in human liver allografts. (A) Dot plot illustrating cellular interactions between CD8+ MAIT cells and myeloid subsets in non‐rejection (NR) vs. rejection (R) samples. (B) Violin plots showing MAIT1 signature scores across four clusters. [file CPR-9999-e70194-s023.jpg]

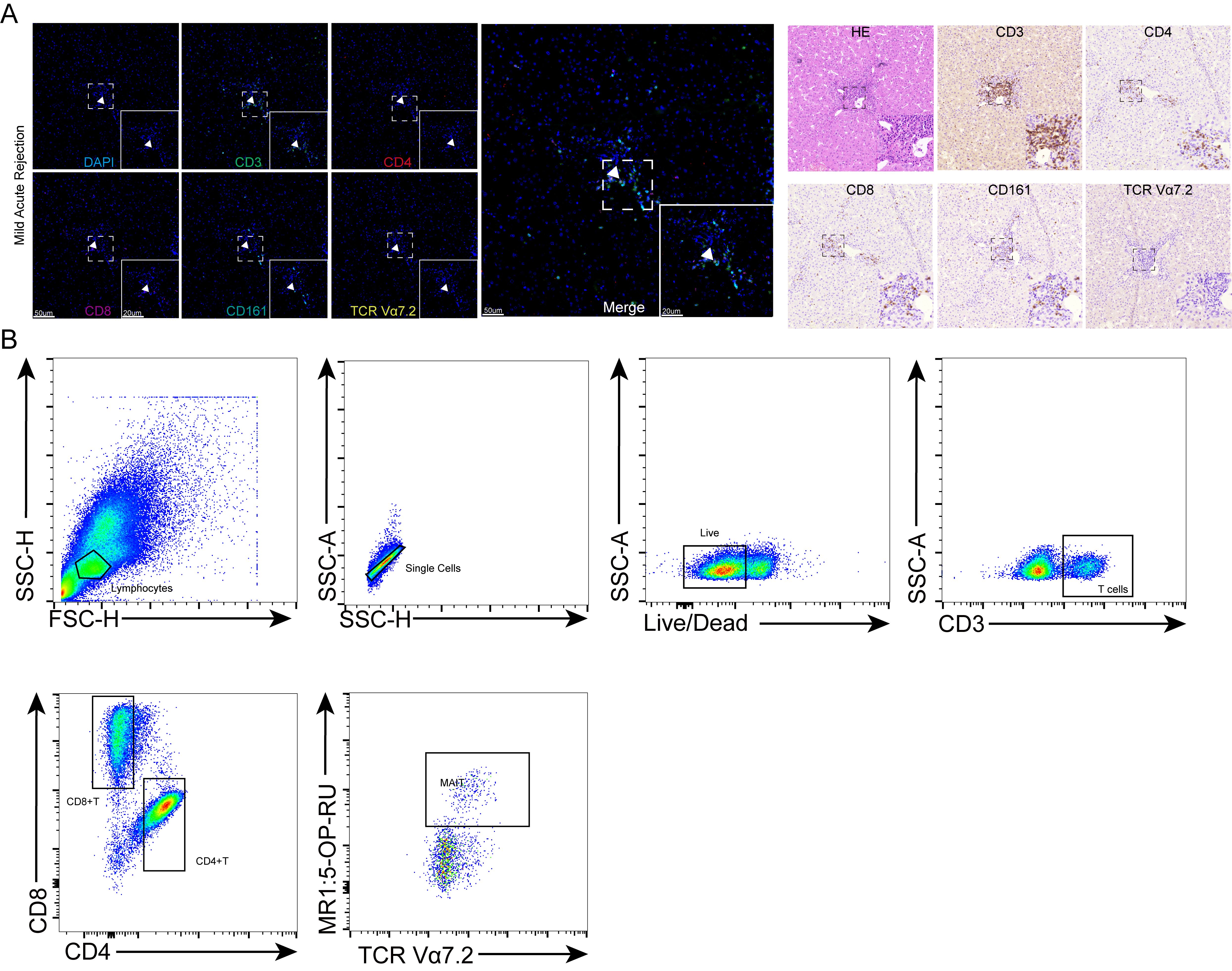

Supplement: Supplementary file 2 — Figure S2: Multiplex immunohistochemical (mIHC) analysis of MAIT cells. Representative mIHC images of mild acute rejection (mAR) and chronic rejection (CR) liver tissues. Staining: DAPI (blue), CD3 (green), CD4 (red), CD8 (pink), CD161 (cyan), and TCR Vα7.2 (yellow). Arrows indicate CD8 + CD161 + TCR Vα7.2+ MAIT cells. Scale bars: 50 μm (main), 20 μm (insets). [file CPR-9999-e70194-s013.jpg]

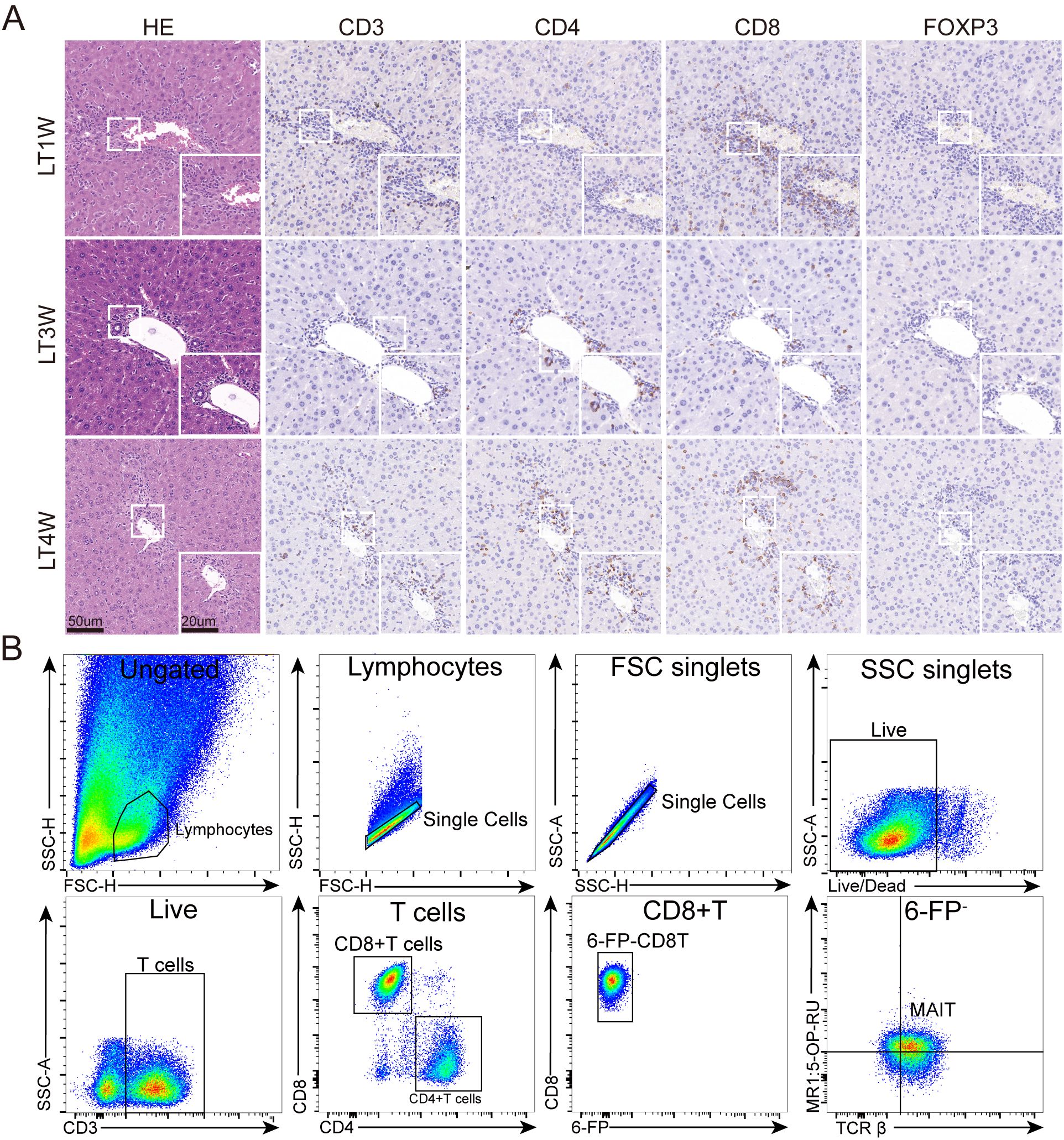

Supplement: Supplementary file 3 — Figure S3: Histological assessment and flow cytometry gating strategy. (A) H&E and IHC staining (CD3, CD4, CD8, FOXP3) of liver tissues at 1, 3, and 4 weeks post‐transplantation (LT1W–LT4W). (B) Flow cytometry gating strategy for identifying CD8+ MAIT cells. [file CPR-9999-e70194-s006.jpg]

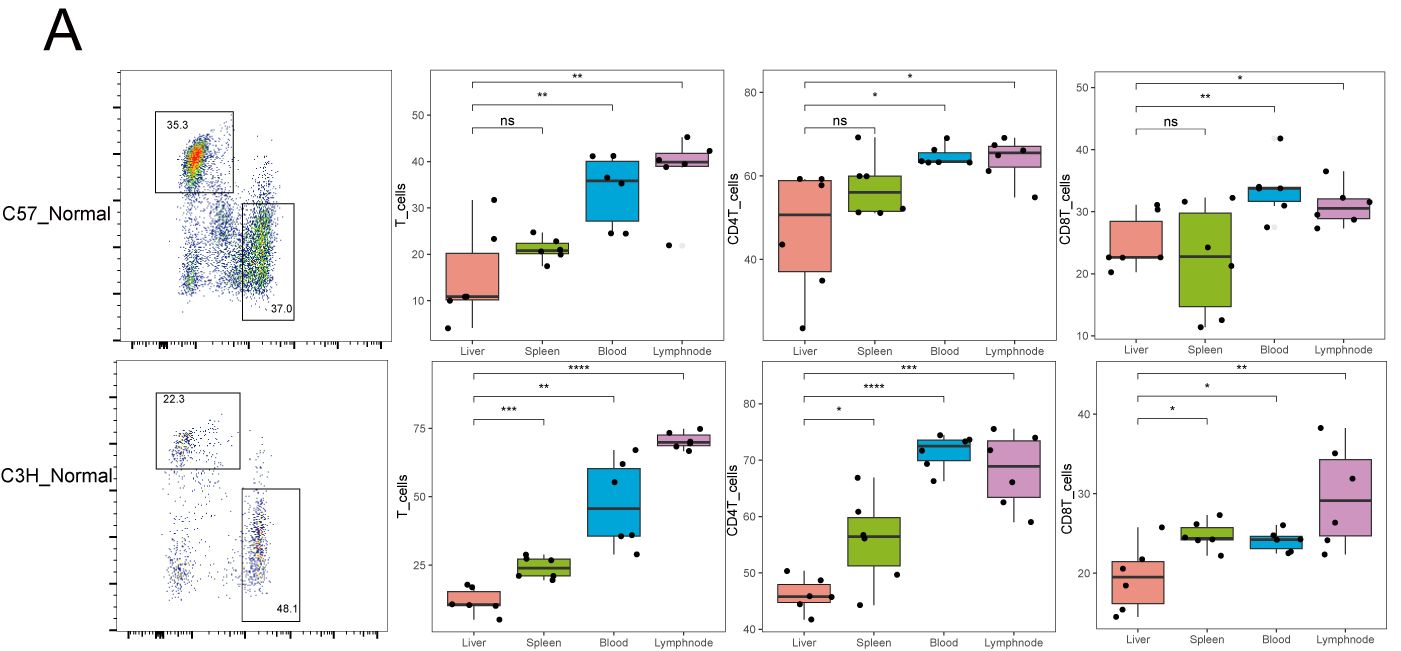

Supplement: Supplementary file 4 — Figure S4: T cell profiling in naïve mice. Flow cytometry analysis of T cell subsets in naïve C57BL/6 and C3H mice. [file CPR-9999-e70194-s016.jpg]

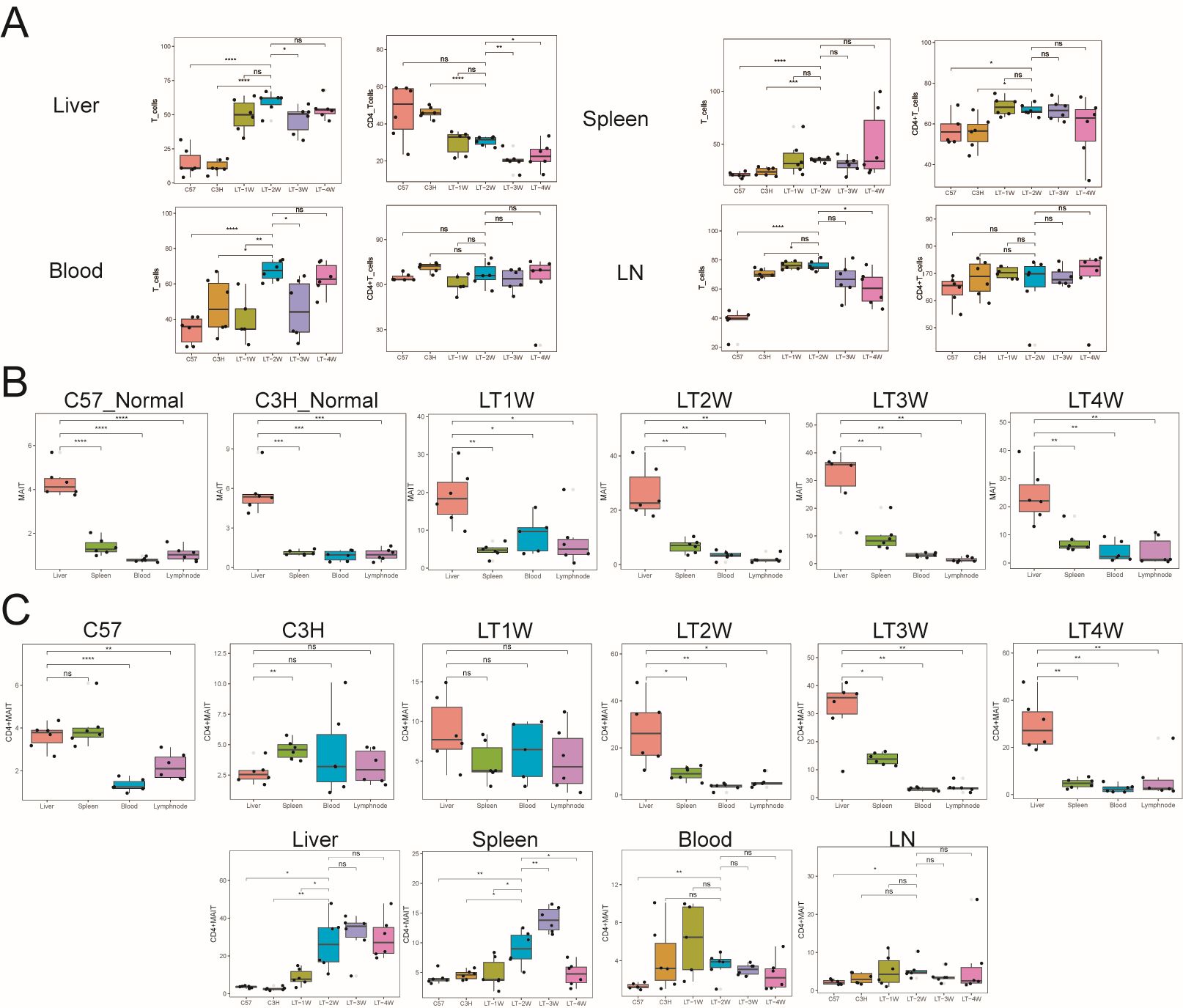

Supplement: Supplementary file 5 — Figure S5: Longitudinal dynamics of T cells and MAIT cells in liver transplantation. (A) Proportions of total T and CD4+ T cells in liver allografts from week 1 to 4 (LT1W–LT4W). (B‐C) Distribution of CD8+ (B) and CD4+ (C) MAIT cells across indicated tissues in control (C57, C3H) and transplant groups over time. [file CPR-9999-e70194-s007.jpg]

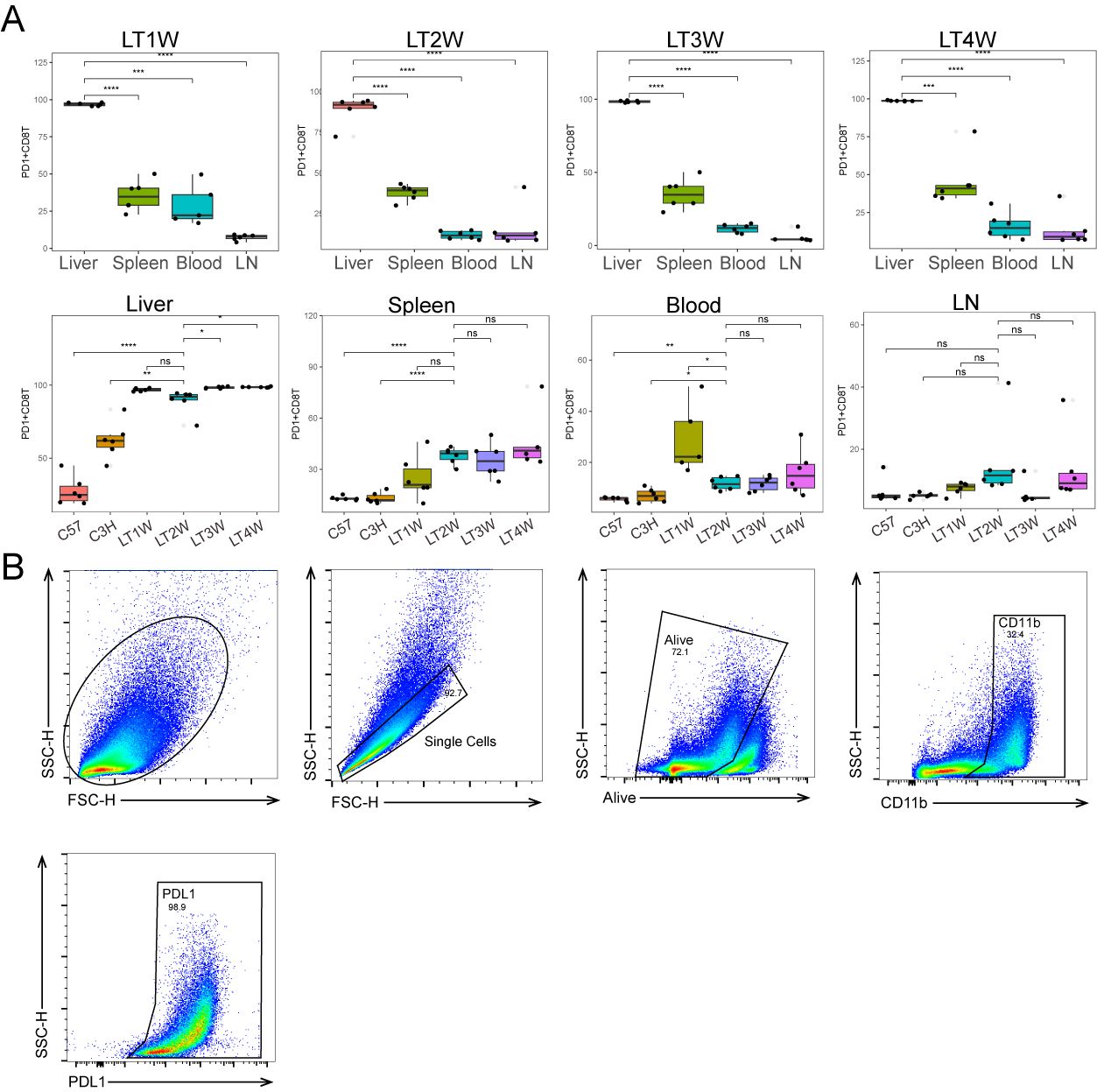

Supplement: Supplementary file 6 — Figure S6: PD‐1 expression kinetics and myeloid gating strategy. (A) Frequency of PD‐1 + CD8+ T cells across tissues at different post‐transplant time points. (B) Flow cytometry gating strategy for PD‐L1+ myeloid cells. [file CPR-9999-e70194-s010.jpg]

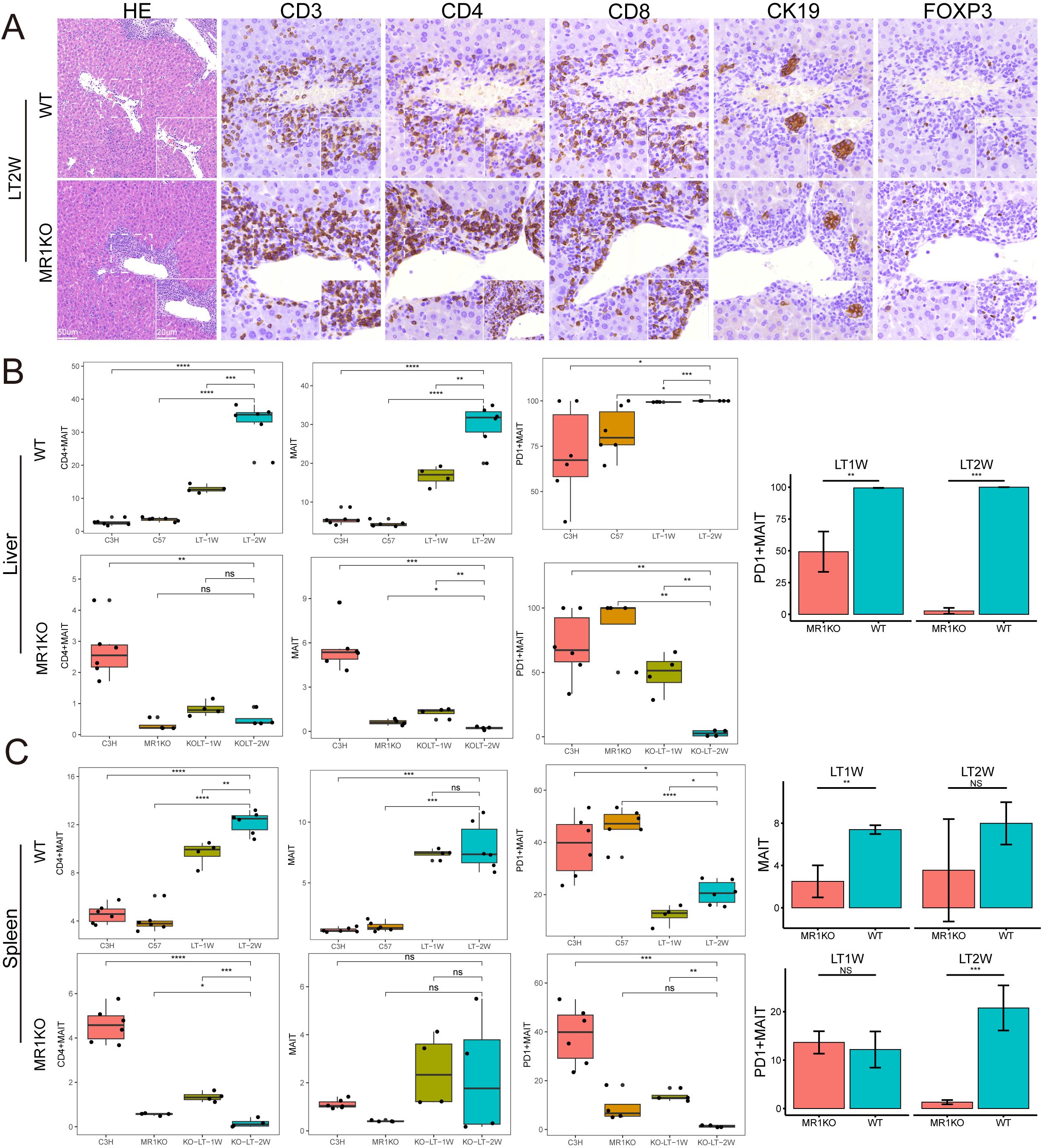

Supplement: Supplementary file 7 — Figure S7: Impact of MR1 deficiency on hepatic T cell subsets. (A) Representative H&E and IHC staining (CD3, CD4, CD8, CK19, FOXP3) of liver allografts at 2 weeks post‐transplant (LT2W). Scale bars: 50 μm (main), 20 μm (insets). (B‐C) Frequencies of CD4+, CD8+, and PD‐1+ MAIT cells in the liver (B) and spleen (C) of WT versus MR1KO recipients over time. [file CPR-9999-e70194-s017.jpg]

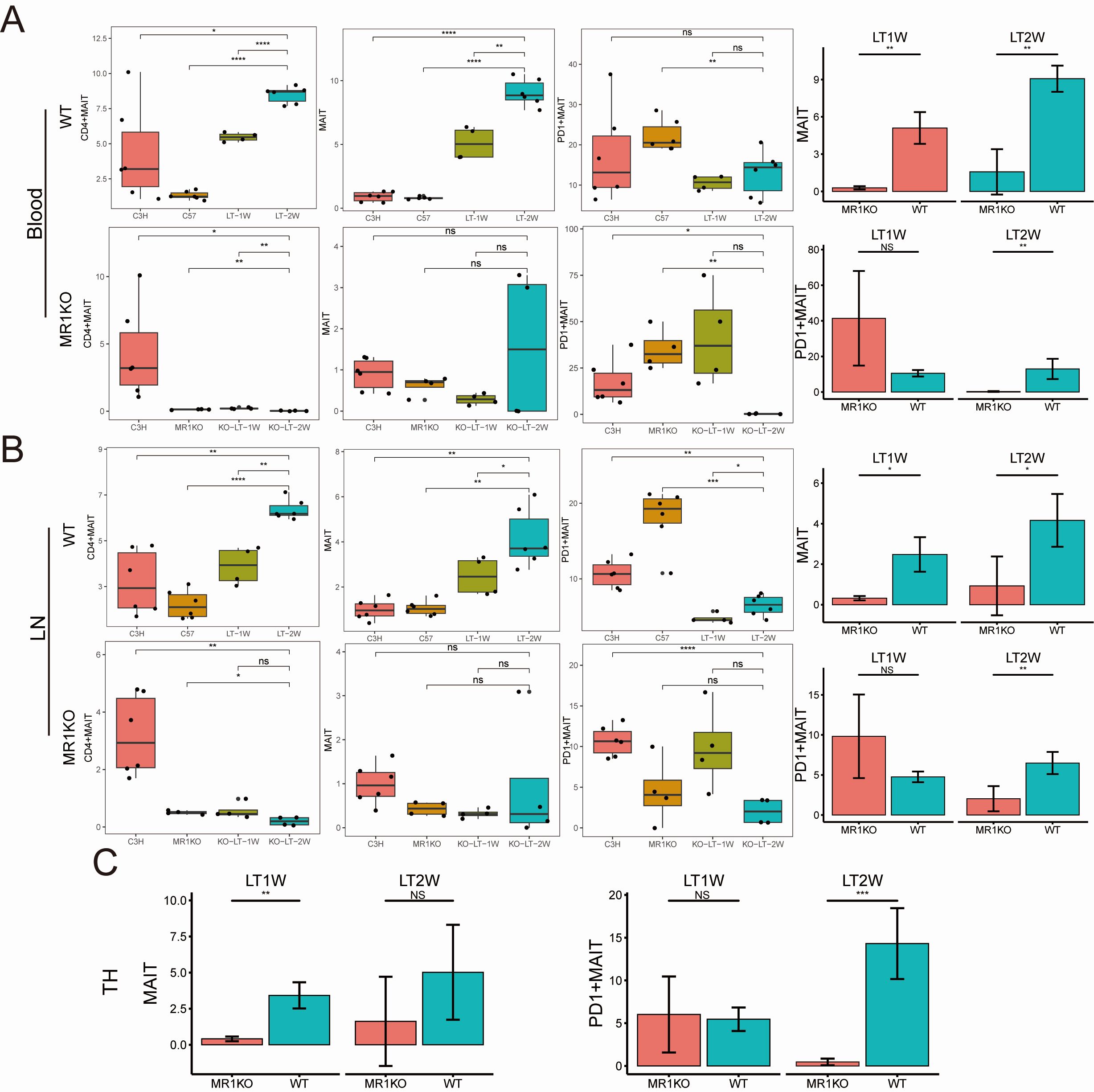

Supplement: Supplementary file 8 — Figure S8: Systemic changes in MAIT cell subsets in MR1KO mice. (A‐B) Frequencies of CD4+, CD8+, and PD‐1+ MAIT cells in peripheral blood (A) and lymph nodes (LN) (B) of WT and MR1KO mice. (C) Comparison of CD8+ and PD‐1+ MAIT cell proportions in the thymus (TH). [file CPR-9999-e70194-s002.jpg]

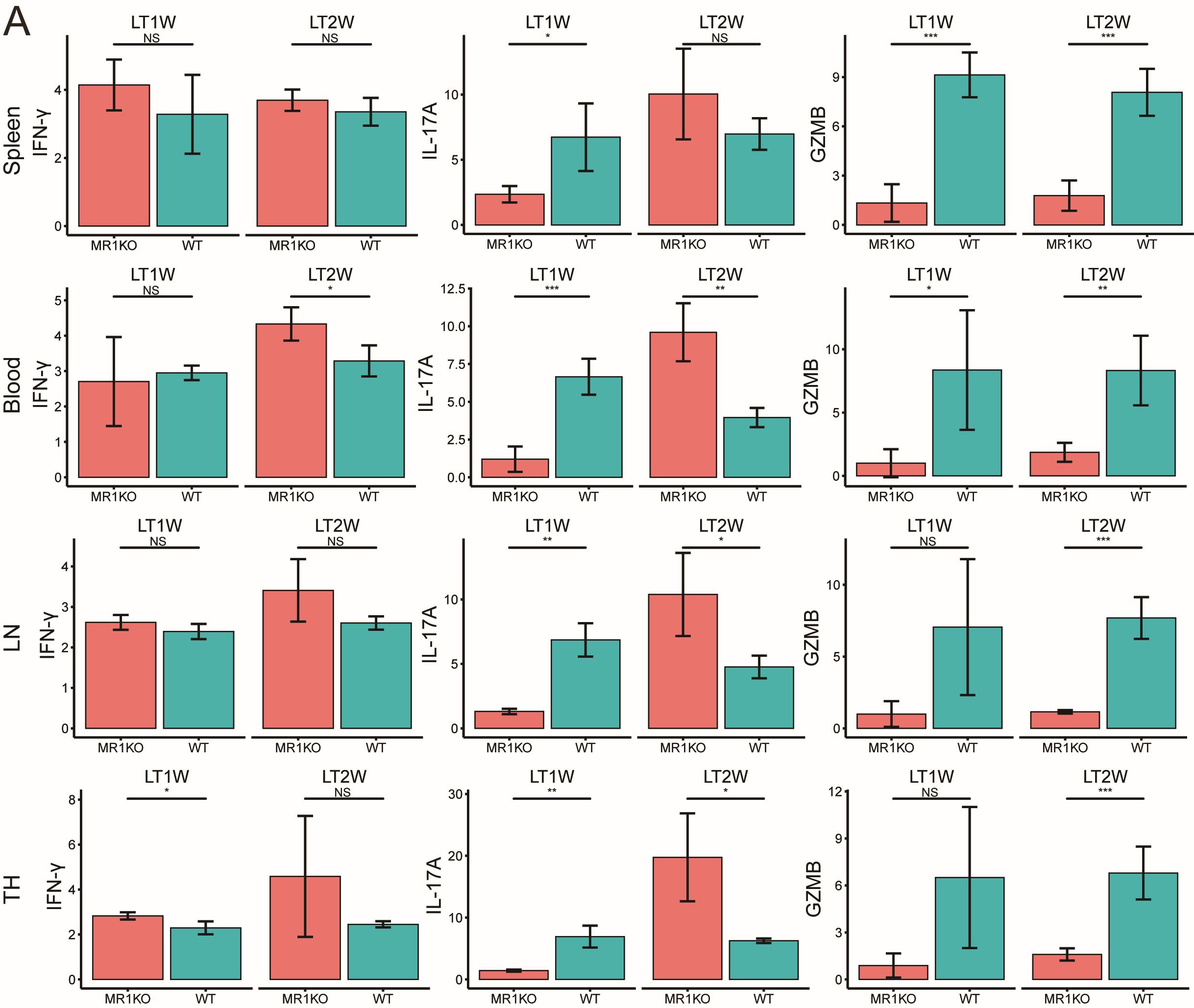

Supplement: Supplementary file 9 — Figure S9: Cytokine and cytotoxicity profiles across tissues. Comparison of IFN‐γ, IL‐17, and Granzyme B (GZMB) expression levels in spleen, blood, lymph nodes, and thymus. [file CPR-9999-e70194-s024.jpg]

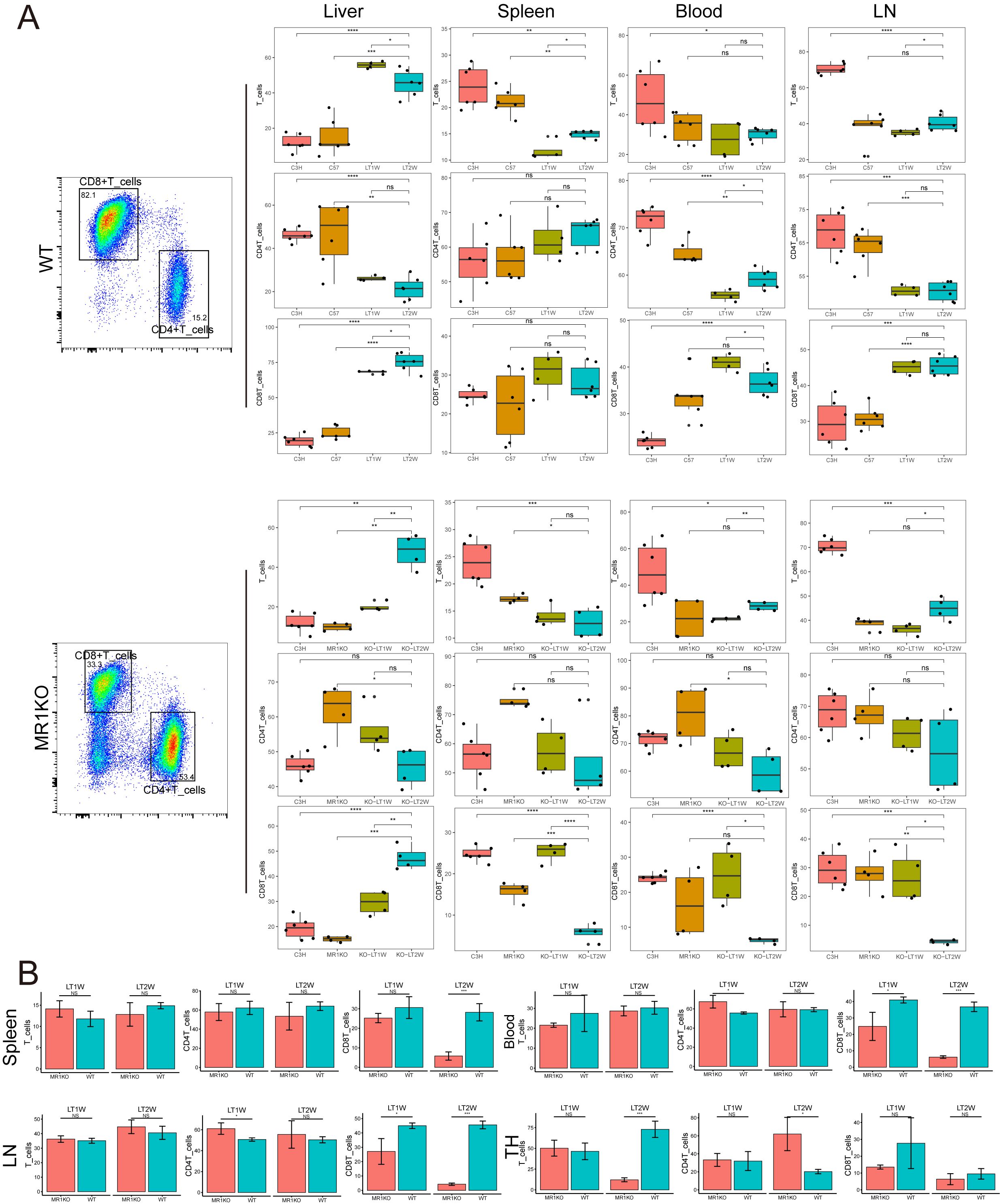

Supplement: Supplementary file 10 — Figure S10: Comparative analysis of T cell compartments in WT and MR1KO mice. (A‐B) Proportions of total T, CD4+ T, and CD8+ T cells in the liver, spleen, blood, and lymph nodes (LN) of WT and MR1KO groups. [file CPR-9999-e70194-s022.jpg]

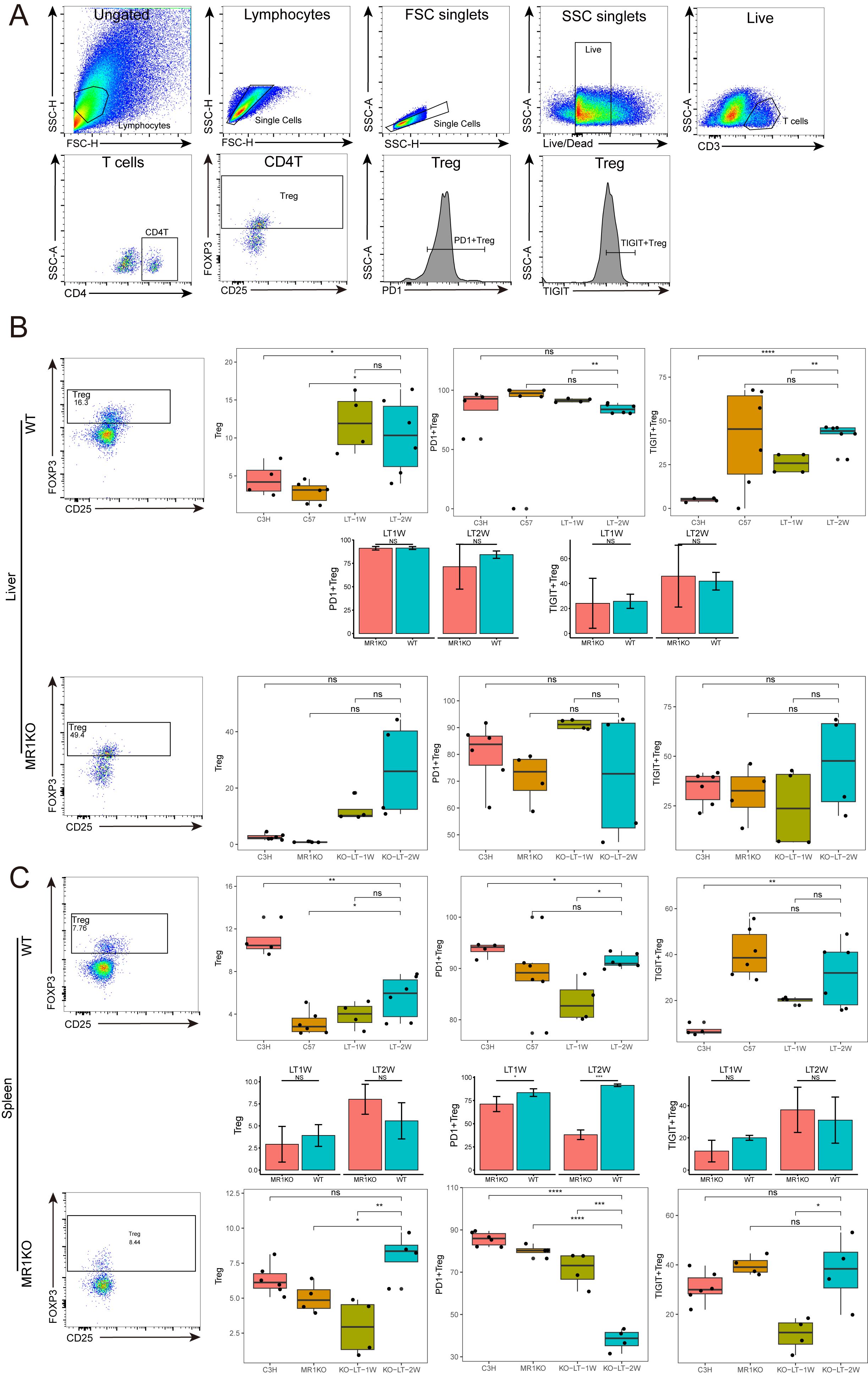

Supplement: Supplementary file 11 — Figure S11: Hepatic and splenic Treg dynamics. (A) Gating strategy for Treg. (B‐C) Temporal changes and comparison of total, PD1+, and TIGIT+ Treg in the liver (B) and spleen (C) of WT and MR1KO mice. [file CPR-9999-e70194-s003.jpg]

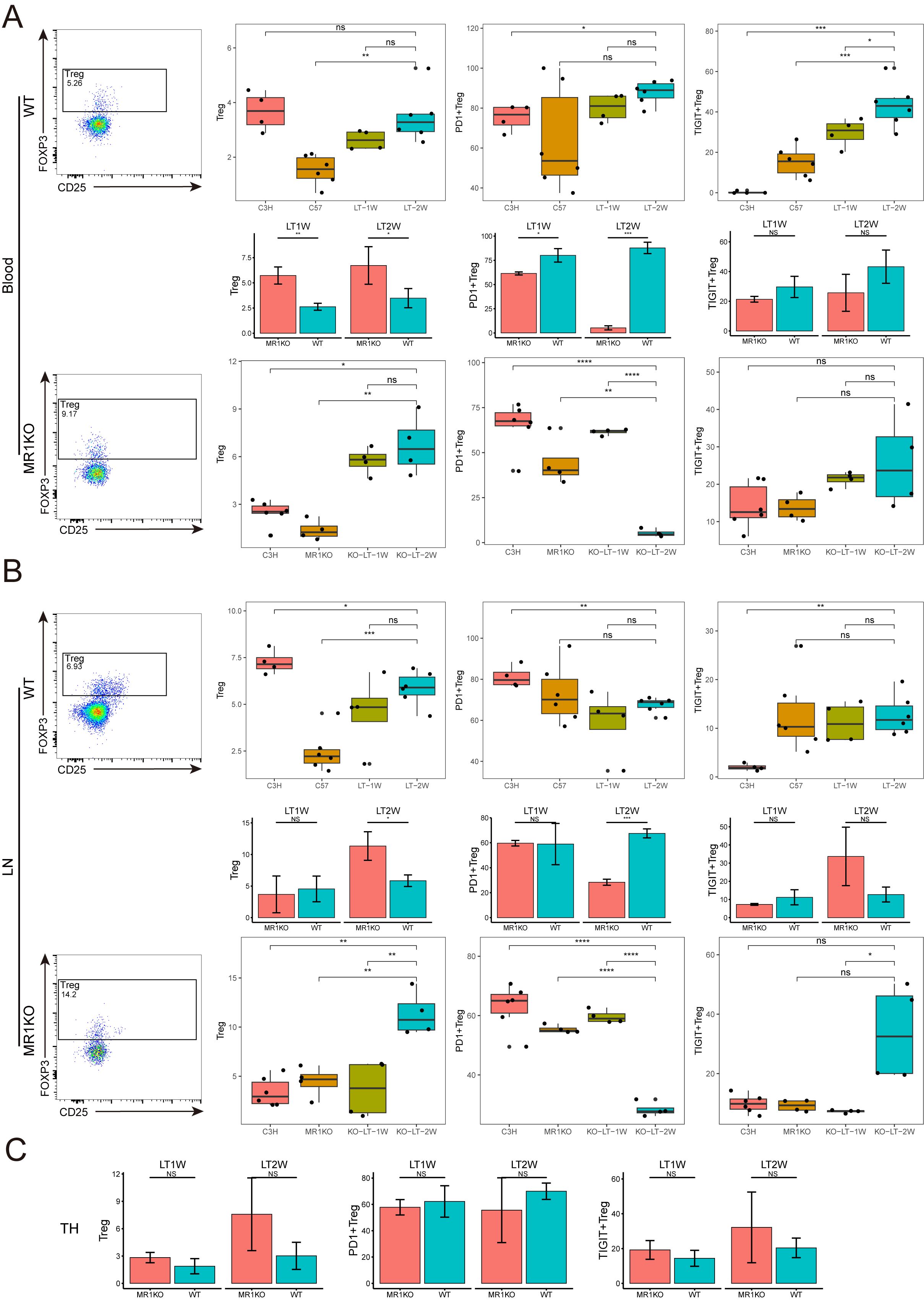

Supplement: Supplementary file 12 — Figure S12: Systemic Treg dynamics in lymphoid tissues. (A‐C) Temporal changes and comparison of total, PD1+, and TIGIT+ Treg in the blood (A), lymph nodes (B), and thymus (C) of WT and MR1KO mice. [file CPR-9999-e70194-s008.jpg]

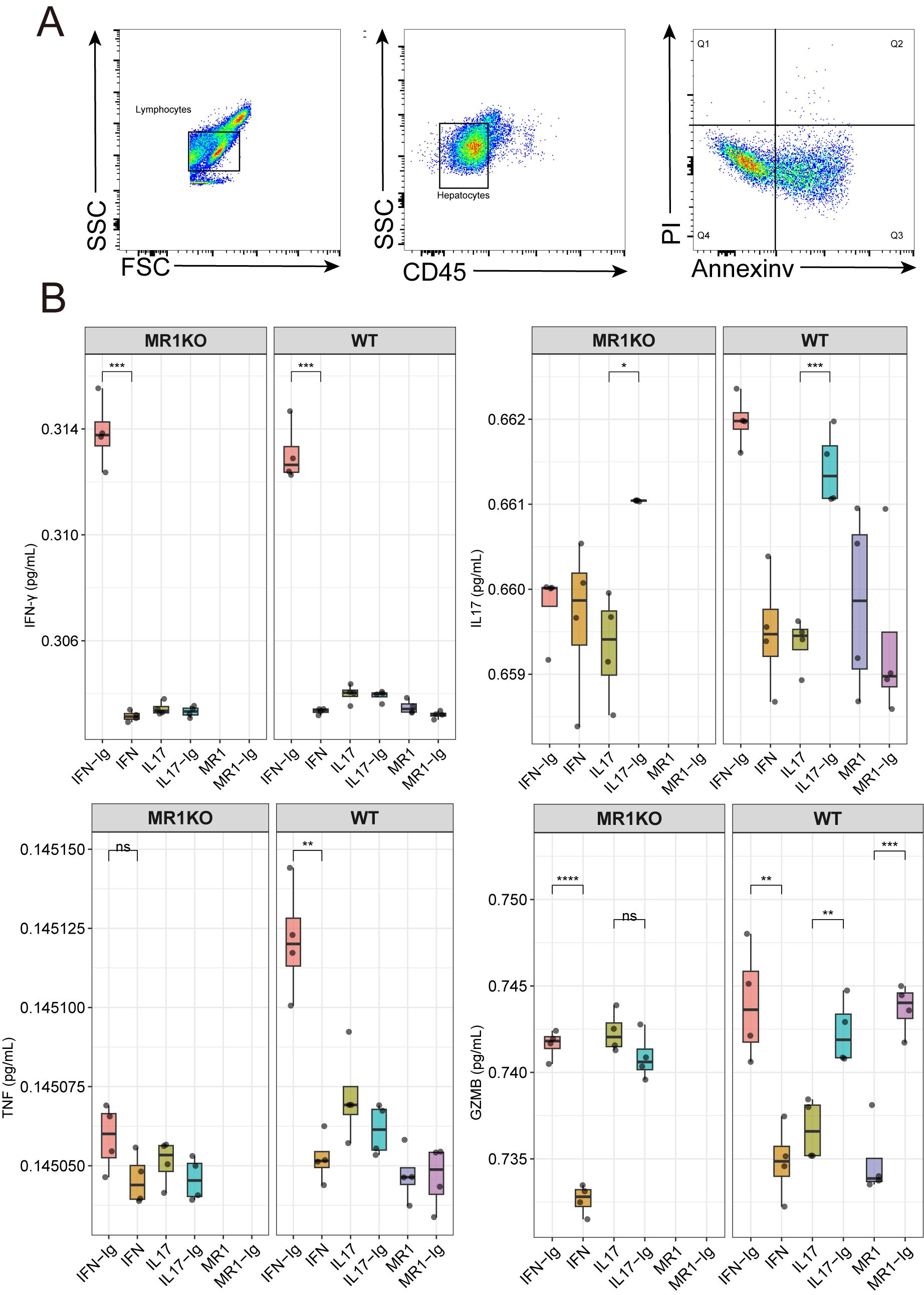

Supplement: Supplementary file 13 — Figure S13: MAIT cell function in intro. (A) Gating strategy for identifying hepatocytes of Annexin V expression. (B) Quantification of cytokine (IFN‐γ, TNF and IL‐17A) and cytotoxic molecule (GZMB) production by co‐culture models in WT and MR1KO. [file CPR-9999-e70194-s019.jpg]

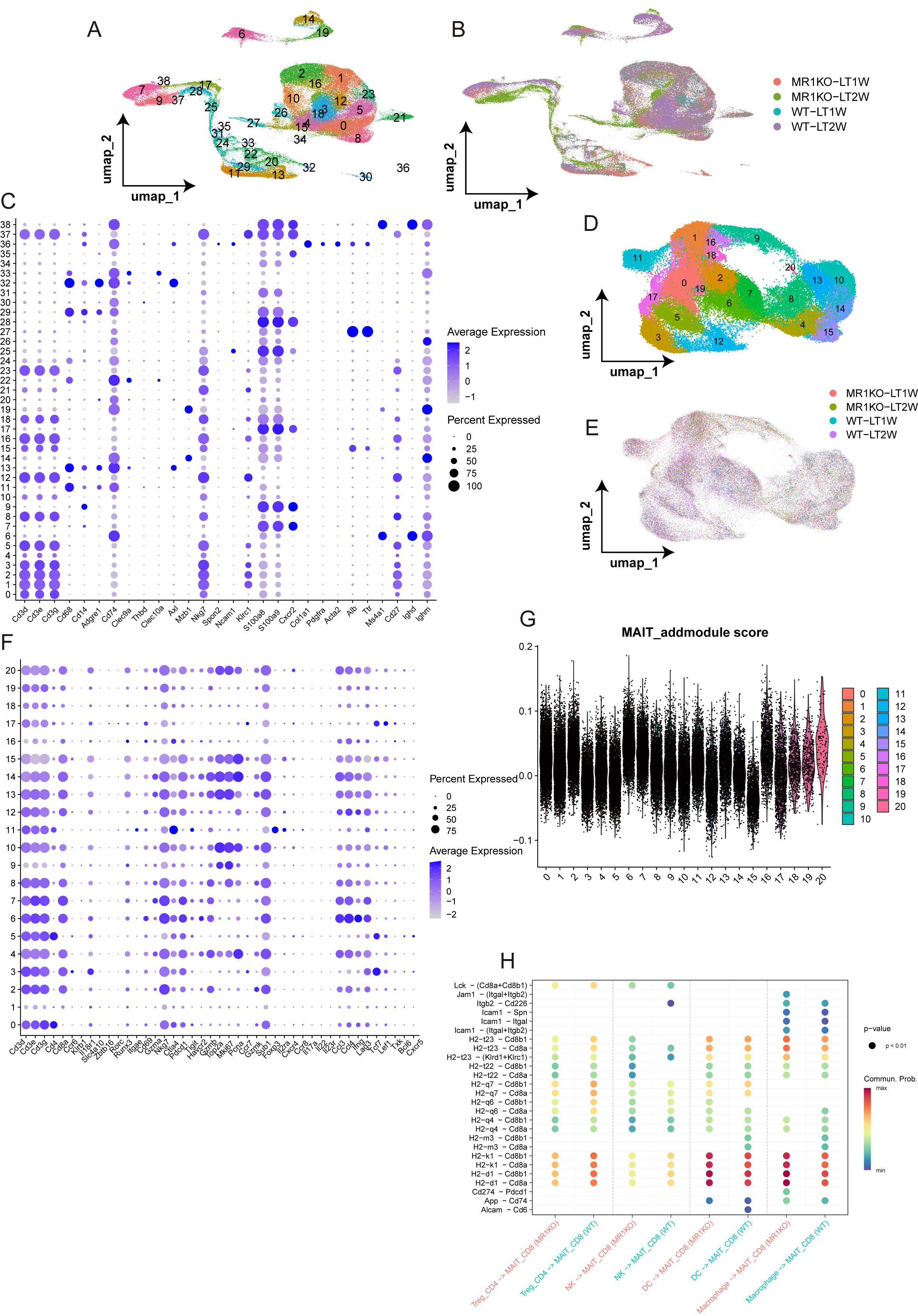

Supplement: Supplementary file 14 — Figure S14: Single‐cell landscape of murine liver allografts. (A‐B) UMAP visualisation of all cell clusters (A) and sample distribution (B‐C) Dot plot of canonical marker genes. (D‐E) UMAP re‐clustering of T cells (D) and sample distribution. (F) Marker genes for T cell subsets. (G) MAIT gene signature scores across clusters. (H) Ligand‐receptor interactions between MAIT cells and macrophages. [file CPR-9999-e70194-s015.jpg]

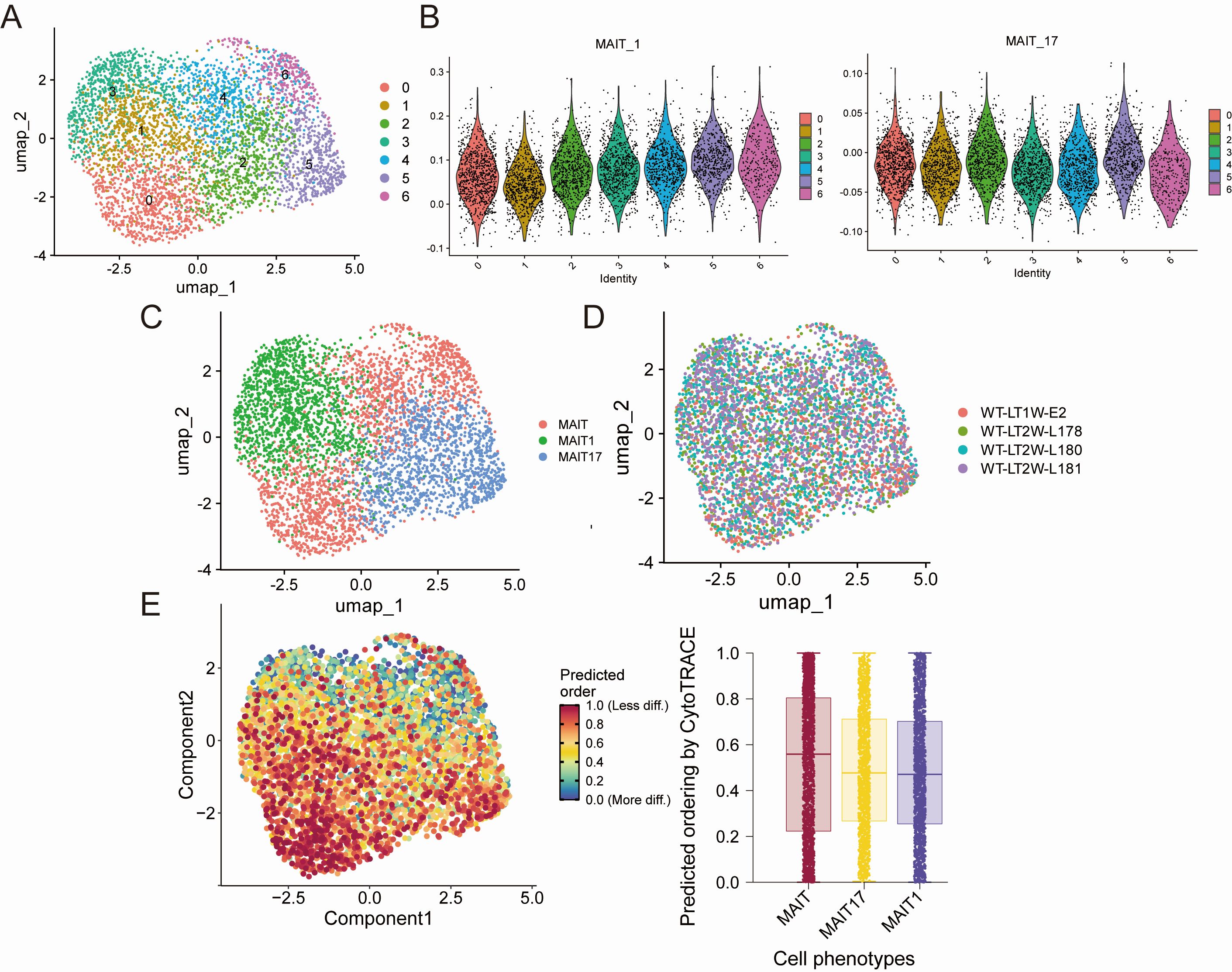

Supplement: Supplementary file 15 — Figure S15: Single‐cell landscape of MAIT cells in murine liver allografts. (A) UMAP visualisation of CD8 + MAIT cell clusters. (B) VlnPlot shows the Addmoudule Score of MAIT1 and MAIT17. (C) UMAP visualisation of annotated CD8 + MAIT cells. (D) UMAP shows the distribution of CD8 + MAIT cells in samples. (E) UMAP projection and box plot show CD8 + MAIT cells were predicted with differentiation states using CytoTRACE. [file CPR-9999-e70194-s009.jpg]

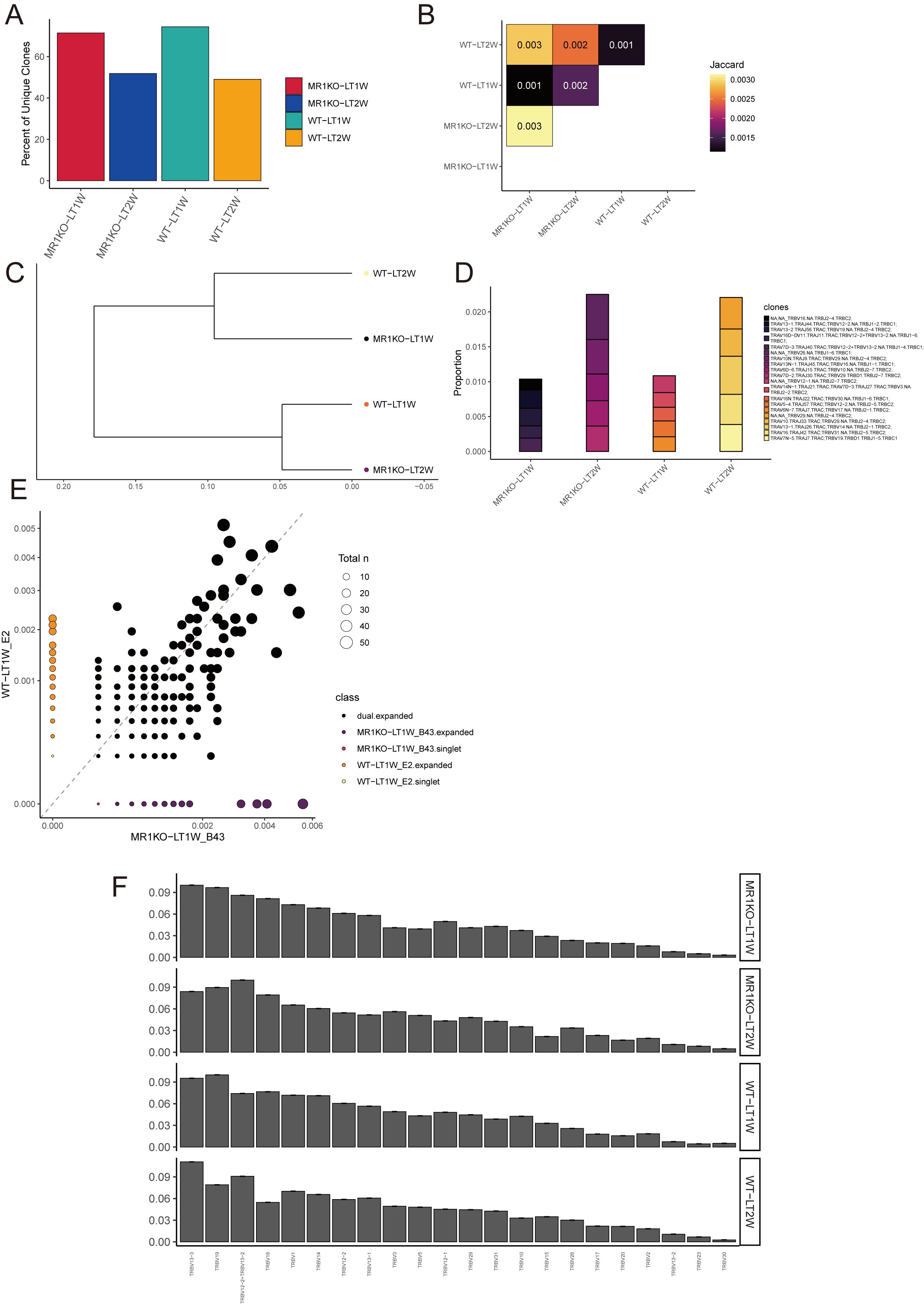

Supplement: Supplementary file 16 — Figure S16: TCR repertoire analysis. (A) Percentage of unique TCR clones per group. (B) Heatmap of Jaccard similarity coefficients between genotypes. (C) Hierarchical clustering based on clonal characteristics. (D) Clonal expansion proportions within each group. (E) Scatter plot comparing clonal frequencies between MR1KO‐LT1W and WT‐LT1W. (F) Clonal size distribution histograms. [file CPR-9999-e70194-s005.jpg]
