# Supplementary material for: Single Cell Multi‐Omics Revealing the Important Role of MR1 Mediated MAIT Cells in Maintaining Rejection for Liver Transplantation
Source: Cell Prolif. 2026 Mar 10:e70194. Online ahead of print. doi: 10.1111/cpr.70194 (PMC13325667; doi:10.1111/cpr.70194)
Supplement: Supplementary file 24 — Data S1: Supporting Information. [file CPR-9999-e70194-s021.docx]

**Materials and Methods**

**Tissue dissociation and preparation**

Fresh tissues were stored in sCelLive™ Tissue Preservation Solution (Singleron) on ice within 30 minutes post-surgery. Specimens were washed three times with Hanks Balanced Salt Solution (HBSS), minced into small fragments, and digested with 3 mL sCelLive™ Tissue Dissociation Solution (Singleron) using the Singleron PythoN™ Tissue Dissociation System at 37 °C for 15 minutes. The cell suspension was collected, filtered through a 40-μm sterile strainer, and treated with GEXSCOPE® Red Blood Cell Lysis Buffer (RCLB, Singleron) at a cell:RCLB volume ratio of 1:2. After incubation at room temperature for 5–8 minutes to lyse red blood cells, the mixture was centrifuged at 300 × g for 5 minutes at 4 °C. The supernatant was discarded, and the cell pellet was resuspended in PBS.

**Library Construction**

Single-cell suspensions (2×10⁵ cells/mL in PBS, HyClone) were loaded onto a microwell chip using the Singleron Matrix® Single Cell Processing System. Barcoding Beads were collected from the chip, followed by reverse transcription of mRNA captured by the beads to generate cDNA, which was amplified via PCR. The amplified cDNA was fragmented, ligated with sequencing adapters, and used to construct scRNA-seq libraries following the GEXSCOPE® Single Cell RNA Library Kits (Singleron) protocol^1^. Individual libraries were diluted to 4 nM, pooled, and sequenced on an Illumina NovaSeq 6000 platform with 150 bp paired-end reads.

**Primary analysis of raw read data**

Raw scRNA-seq reads were processed using the CeleScope (v1.9.0) pipeline (<https://github.com/singleron-RD/CeleScope>). Briefly, Cutadapt v1.17^2^ was used to trim low-quality reads, poly-A tails, and adapter sequences, followed by extraction of cell barcodes and UMIs. Reads were mapped to the GRCm38 reference genome (Ensembl version 92 annotation) using STAR v2.6.1a. UMI and gene counts per cell were generated with featureCounts v2.0.1 to construct expression matrix files for downstream analysis.

**Single-Cell Gene Expression and Subcluster Delineation**

The single-cell data analysis of humans was based on our previous research^3,4^. Single-cell RNA-seq data were analyzed using the Seurat R package (version 5.1.0) ^5^. Quality control was performed by filtering out cells expressing fewer than 200 genes or exhibiting mitochondrial transcript proportions greater than 10% to exclude low-quality cells and doublets. Following normalization, batch effects across samples were corrected using the Canonical Correlation Analysis (CCA) integration method embedded in Seurat to align shared biological states. Highly variable features were identified via FindVariableFeatures, followed by principal component analysis (PCA) on scaled data. To specifically characterize MAIT cells, we utilized a defined MAIT gene signature derived from Garner et al.^6^, and cell scores were calculated using the AddModuleScore function to guide annotation. Dimensionality reduction and clustering were performed using FindNeighbors (dimensions 1–10) and FindClusters (resolution 1.0). Uniform manifold approximation and projection (UMAP) was applied for data visualization.

**Cell Type Determination**

We identified differentially expressed features across cell clusters using the FindMarkers and FindAllMarkers functions. Cell types were then annotated by referencing known biological categories and a curated set of canonical marker genes^7^.

**Functional Enrichment Analysis and MAIT Scores**

After annotating each cell type, we performed functional enrichment analysis on the differentially expressed genes across cell clusters. This analysis, aimed at uncovering the biological processes and potential functions of specific cell types, was based on GO and KEGG pathways. The analysis was conducted using the clusterProfiler package (version 3.17.0) along with the org.Mm.eg.db package (version 3.21.0). To ensure the significance of our findings, a p-value cutoff of 0.05 was applied for both GO and KEGG analysis. The top ten terms from the results were visualized using barplots, providing a clear and concise graphical summary of the key functional characteristics associated with each cell type.

To calculate MAIT scores using gene set analysis, we employed the AddModuleScore function in the Seurat package, leveraging MAIT-related marker genes^6^. This method applied specific gene sets of interest obtained from previously published studies or datasets. A score was computed for each cell based on the expression levels of genes within each set, allowing for a detailed, cell-specific analysis of gene expression patterns.

**Cell-Cell Communication Analysis**

To analyze intercellular interactions, we utilized the CellChat package (version 1.6.1)^8^, focusing on ligand-receptor interactions informed by the KEGG signaling pathway database and recent experimental findings. The process began with identifying differentially expressed signaling genes to highlight those showing significant expression variations. Next, we calculated the ensemble average expression to provide an overview of gene expression patterns across cell types. Finally, we assessed the probability of intercellular communication, offering key insights into the complex signaling networks and interactions among the diverse cell populations in our study.

**Pseudotime Analysis**

We performed trajectory analysis using the CytoTRACE package (version 0.3.3)^9^, allowing us to track the developmental progression of cells in a pseudo-temporal order. This analysis encompassed CD8+MAIT cells in human and mouse. CytoTRACE scores range from 0 to 1, while higher scores indicate higher stemness (less differentiation) and vice versa.

**TCR Sequencing Analysis**

TCR-α/β sequencing data were merged into the Seurat metadata for various integrated analyses using scRepertoire package (version 2.2.1)^10^.

**Bulk RNA Sequencing Analysis**

To validate our findings, we obtained transcriptome data from the NCBI Gene Expression Omnibus (GEO). Specifically, we accessed datasets GSE145780^11^, which contain gene expression profiles derived from microarray analysis of liver transplant biopsies. These datasets included samples from both rejection and non-rejection cases, offering a comprehensive perspective on gene expression changes associated with different transplant outcomes.

**Histological Staining**

Fresh liver tissue samples were fixed in 4% paraformaldehyde and embedded in paraffin before being sectioned into 4 μm slices. Hematoxylin and eosin (H&E) staining was performed for morphological evaluation, while reticular fiber staining was used to assess tissue architecture. To identify and highlight fibrotic regions within the liver tissue, Masson’s trichrome staining was also applied.

**IHC Staining Analysis**

IHC staining was performed on liver tissue sections to detect specific antigens, including CD3, CD4, CD8A, CD161, TCR Vα7.2, all sourced from Abcam. The tissue slides were first deparaffinized and dehydrated, followed by heat-induced antigen retrieval in Tris-EDTA buffers at pH 6.0 and 9.0. To minimize background interference, 3% hydrogen peroxide (H₂O₂) was used to neutralize endogenous peroxidases, and 3% bovine serum albumin (BSA) was applied to block nonspecific antigens.

The slides were then incubated with primary antibodies targeting the aforementioned antigens. Signal detection was performed using a TSA kit from Nanjing Freethinking Biotechnology Co., Ltd. (China), and nuclei were counterstained with DAPI. Finally, the stained slides were scanned using a Pannoramic MIDI slice scanner (3Dhistech, Hungary), and image analysis was conducted with the HALO 2.0 Area Quantification algorithm (Indica Labs, Corrales, NM) at Nanjing Freethinking Biotechnology Co., Ltd. (China).

**Flow Cytometry**

For flow cytometric analysis, cells were stained with a panel of fluorochrome-conjugated antibodies, including live/dead staining (1:1000, Zombie R718™ Fixable Viability Kit, APCA700), CD3 (1:500, 17A2, PerCP/Cyanine5.5), CD4 (1:500, RM4-5, FITC), CD8 (1:500, 53-6.7, PE/Cyanine7), TCRβ (1:500, H57-597, Brilliant Violet 421), and PD1 (1:500, 29F.1A12, ECD), CD25 (1:500, PC61, APC), FOXP3 (1:250, 150D, PE), PD1 (1:500, 29F.1A12, PECy7), TIGIT (1:500, A15153G, Brilliant Violet 421), IFN-γ (1:500, XMG1.2, Brilliant Violet 650), IL-17A (1:500, TC11-18H10.1, Brilliant Violet 605) and Granzyme B (1:500, QA16A02, APCCy7) all obtained from BioLegend. The MR1 tetramers (1:500, PE, APC) loaded with 5-OP-RU or 6-FP (both tetramers from the NIH Tetramer Core Facility; Emory University, GA). The staining process was performed following the manufacturer’s protocols.

For intracellular staining, fluorochrome-labeled antibodies were added to the cells and incubated in 5% BSA at a room temperature for 30 min. The cells were fixed and permeabilized with Foxp3 Transcription Factor Staining Buffer Set (eBioscience, 00-5523-00) at a room temperature for 30 min for intracellular Foxp3 staining, following the manufacturer’s recommendations.

For intracellular cytokine staining, cells were stimulated with PMA (phorbol 12-myristate-13-acetate)/ionomycin, and protein transport inhibitor (Brefeldin A) for 6 hours at 37°C, 5% CO2 using DMEM/F-12 [(VivaCell, C3130-0500) containing 10% fetal bovine serum (FBS)]. Surface staining was performed at a room temperature for 30 min, and cells were stained for intracellular cytokines using the Intracellular Fixation & Permeabilization Buffer Set (eBioscience) according to the manufacturers’ instructions.

After staining, the samples were using URIT Flow Cytometer, BF-730 B5R3V6, URIT Medical Electronic Co., Ltd., Guilin China and analyzed using FlowJo software (version 10.8.1) to examine and quantify distinct cell populations based on their fluorescence and antibody binding profiles.

**In vitro co-culture and cytokine blockade assay**

Primary hepatocytes were isolated from C57BL/6, MR1KO C57BL/6 and seeded into plates. MAIT cells were stained using MR1:5-OP-RU-PE tetramer and sorted from C3H/He liver using anti-PE microbeads in LS columns (Miltenyi). The two cell types were co-cultured at a ratio of 5:1 and cells were pre-stimulated with 5-OP-RU for 2 hours. To assess the role of cytokines, neutralizing antibodies against MR1 (Clone 26.5,10ng/ml), IL-17A (Clone 17F3, 10ng/ml) and IFN-γ (Clone XMG1.2, 10ng/ml), or isotype controls, were added to the culture. After 24 hours, cytotoxicity was evaluated by Annexin V staining and IFN-γ, IL-17A, TNF-α and GZMB release assay.

**Statistical analysis**

Statistical analysis and data visualization were conducted using R software (version 4.4.3). Results are expressed as mean ± standard error of the mean (SEM). An unpaired Student’s t-test was employed to evaluate statistical significance between two groups, correlation analysis was assessed by Spearman’s rank correlation with significance levels denoted as *P < 0.05, **P < 0.01, and ***P < 0.001.

**Acknowledgements**

We thank NIH Tetramer Core Facility for providing us mMR1-5-OP-RU Tetramer and mMR1-6-FP Tetramer. We appreciate the help and support of Li Bai from the School of Basic Medical Sciences, University of Science and Technology of China.

**References**

1. Jiang P, Luo L, Li X, et al. PTX3 exacerbates hepatocyte pyroptosis in hepatic ischemia-reperfusion injury by promoting macrophage M1 polarization. Int Immunopharmacol. 2024;143(Pt 3):113604. doi:10.1016/j.intimp.2024.113604

2. Zhou LX, Jiang YZ, Li XQ, et al. Myeloid-derived suppressor cells-induced exhaustion of CD8 + T-cell participates in rejection after liver transplantation. Cell Death Dis. 2024;15(7):507. doi:10.1038/s41419-024-06834-z

3. Li X, Li S, Wu B, et al. Landscape of Immune Cells Heterogeneity in Liver Transplantation by Single-Cell RNA Sequencing Analysis. Front Immunol. 2022;13:890019. doi:10.3389/fimmu.2022.890019

4. Li X, Li R, Miao X, et al. Integrated Single Cell Analysis Reveals An Atlas of Tumor Associated Macrophages in Hepatocellular Carcinoma. Inflammation. 2024;47(6):2077-2093. doi:10.1007/s10753-024-02026-1

5. Wu T, Hu E, Xu S, et al. clusterProfiler 4.0: A universal enrichment tool for interpreting omics data. The Innovation. 2021;2(3):100141. doi:10.1016/j.xinn.2021.100141

6. Single-cell diversity and functional plasticity of human MAIT cells. Nat Immunol. 2023;24(9):1409-1410. doi:10.1038/s41590-023-01600-3

7. Li X, Li S, Wang Y, et al. Single cell RNA-sequencing delineates CD8+ tissue resident memory T cells maintaining rejection in liver transplantation. 2024;14(12).

8. Jin S, Guerrero-Juarez CF, Zhang L, et al. Inference and analysis of cell-cell communication using CellChat. Nat Commun. 2021;12(1):1088. doi:10.1038/s41467-021-21246-9

9. Gulati GS, Sikandar SS, Wesche DJ, et al. Single-cell transcriptional diversity is a hallmark of developmental potential. Science. 2020;367(6476):405-411. doi:10.1126/science.aax0249

10. Borcherding N, Bormann NL, Kraus G. scRepertoire: An R-based toolkit for single-cell immune receptor analysis. F1000Res. 2020;9:47. doi:10.12688/f1000research.22139.2

11. Madill-Thomsen K, Abouljoud M, Bhati C, et al. The molecular diagnosis of rejection in liver transplant biopsies: First results of the INTERLIVER study. American Journal of Transplantation. 2020;20(8):2156-2172. doi:10.1111/ajt.15828
